# Predicting Consumer Biomass, Size-Structure, Production, Catch Potential, Responses to Fishing and Associated Uncertainties in the World’s Marine Ecosystems

**DOI:** 10.1371/journal.pone.0133794

**Published:** 2015-07-30

**Authors:** Simon Jennings, Kate Collingridge

**Affiliations:** Centre for Environment, Fisheries and Aquaculture Science, Lowestoft, NR33 0HT, United Kingdom; Technical University of Denmark, DENMARK

## Abstract

Existing estimates of fish and consumer biomass in the world’s oceans are disparate. This creates uncertainty about the roles of fish and other consumers in biogeochemical cycles and ecosystem processes, the extent of human and environmental impacts and fishery potential. We develop and use a size-based macroecological model to assess the effects of parameter uncertainty on predicted consumer biomass, production and distribution. Resulting uncertainty is large (e.g. median global biomass 4.9 billion tonnes for consumers weighing 1 g to 1000 kg; 50% uncertainty intervals of 2 to 10.4 billion tonnes; 90% uncertainty intervals of 0.3 to 26.1 billion tonnes) and driven primarily by uncertainty in trophic transfer efficiency and its relationship with predator-prey body mass ratios. Even the upper uncertainty intervals for global predictions of consumer biomass demonstrate the remarkable scarcity of marine consumers, with less than one part in 30 million by volume of the global oceans comprising tissue of macroscopic animals. Thus the apparently high densities of marine life seen in surface and coastal waters and frequently visited abundance hotspots will likely give many in society a false impression of the abundance of marine animals. Unexploited baseline biomass predictions from the simple macroecological model were used to calibrate a more complex size- and trait-based model to estimate fisheries yield and impacts. Yields are highly dependent on baseline biomass and fisheries selectivity. Predicted global sustainable fisheries yield increases ≈4 fold when smaller individuals (< 20 cm from species of maximum mass < 1kg) are targeted in all oceans, but the predicted yields would rarely be accessible in practice and this fishing strategy leads to the collapse of larger species if fishing mortality rates on different size classes cannot be decoupled. Our analyses show that models with minimal parameter demands that are based on a few established ecological principles can support equitable analysis and comparison of diverse ecosystems. The analyses provide insights into the effects of parameter uncertainty on global biomass and production estimates, which have yet to be achieved with complex models, and will therefore help to highlight priorities for future research and data collection. However, the focus on simple model structures and global processes means that non-phytoplankton primary production and several groups, structures and processes of ecological and conservation interest are not represented. Consequently, our simple models become increasingly less useful than more complex alternatives when addressing questions about food web structure and function, biodiversity, resilience and human impacts at smaller scales and for areas closer to coasts.

## Introduction

Estimates of the abundance, production, distribution and size-structure of marine consumers are needed to determine contributions to biogeochemical cycles and ecosystem function [1, 2], human and environmental impacts on populations, food webs and ecosystems [3, 4, 5] and fishery potential [6–8]. Obtaining estimates, and quantifying uncertainties, remains a challenge; especially when large areas of ocean and many species are studied rarely, if at all [9]. In shelf seas close to relatively wealthy nations, where many species have economic or conservation value, regional estimates may be good [10]. But, for a range of purposes, and especially the global analysis of biogeochemical processes and assessment of human and environmental impacts, greater spatial coverage is needed.

A variety of modelling approaches are employed to estimate abundance and distribution of marine species or communities on global scales [1, 4–8, 10–13]. For fish, which often dominate the abundance of consumers with intermediate body sizes (range 1 g to 1000 kg; the focus of this paper), two existing point estimates of biomass based on a size-based [11] and box model [1] are 9×10^8^ t and 2×10^9^ t respectively. Both were recently challenged as too low following analysis of acoustic data from a transect spanning the tropical and sub-tropical oceans [14]. This interpretation of acoustic data led to median biomass estimates of 11–15 ×10^9^ t for the mesopelagic fishes in the oceanic region from 40°N to 40°S. This was over one order of magnitude higher than a previous biomass estimate for this group of fishes [15] and higher than other global biomass estimates for all fishes [1, 11]. It is challenging to reconcile these estimates, although factors contributing to some differences are clear. For example, assumed or reported differences in transfer efficiency, especially in the first step of the food chain [8, 11, 16].

Potential fisheries production has been estimated with mechanistic and statistical models. Ryther [6], based on assumed food chains lengths and transfer efficiencies in different oceanic regions, estimated total fish production of 240 × 10^6^ t yr^-1^. He predicted that 100 × 10^6^ t yr^-1^ of this production could be caught sustainably by fisheries. This was half an earlier estimate of 200 × 10^6^ t yr^-1^ [17]. Statistical relationships between primary production or chlorophyll concentration and fisheries yield have since been explored at regional and global scales. Within regions these variables may be correlated with yield [18, 19], but correlations tend to weaken or break down at global scales [20, 21]. This is expected when realised yield is not a consistent indicator of potential yield, because the former depends on fishery history and management, relative fishing intensity and the species, sizes and hence trophic levels targeted and selected. Few of these factors are consistent among marine ecosystems [22] and cannot be accounted for with available data because catch statistics rarely include both size and species composition. Closer correlations between yield and chlorophyll or primary production, within ecosystems, may reflect greater similarities in fisheries and their management at this scale [18, 19, 22]. Further, some of the variance in the relationship between primary production and yield among ecosystems is consistent with the complex relationship between primary production and production at higher trophic levels; which needs to account for differences in energy transfer from primary producers. These result from changes in particle-export ratios and the ratio of secondary to primary production [21]. To assess potential yield, an alternate approach is to model the fish production process and the effects of fishing. If assumed fisheries size selection and relative fishing mortality are consistent among regions then estimates of potential yield are comparable.

Primary production of 20 × 10^9^ t C yr^-1^ was assumed by Ryther [6] when estimating global fish production, less than half the current estimates based on ocean-colour data and general circulation models (GCM) [23]. In a model comparison exercise for 1998 [23], the majority of models predicted global primary production of 49 to 60 × 10^9^ t C yr^-1^, with the mean for ocean-colour models slightly lower than for GCM. GCM tend to estimate higher primary production than ocean-colour based models in the Southern Ocean, at low temperatures and in the equatorial band, but estimate lower primary production in eutrophic regions [23].

Locally and regionally, the abundance, production, distribution and size-structure of consumers can be measured directly and/ or estimated with relatively complex models. Models are often structured and parameterised around species and species-groups, and results can be compiled to provide large-scale and even near-global assessments [4, 16]. However, models are not available for all regions and the range of parameterisations and structures limit the systematic and equitable assessment of outputs and uncertainty. These tailored and data-intensive models are effectively complemented by models with lower parameter demands. Such models can be applied in a systematic way in all locations to comprehensively assess the effects of parameter uncertainty and to compare characteristics of different ecosystems.

Modellers have conceptualised food webs by assuming species-, size- or species- and size-based interactions. Size-based approaches account for the substantial role of body size in structuring food webs, which results from the dominance of small primary producers [24], size-based predation [25] and ontogenetic increases in the trophic levels of many consumers [26]; which may grow 5–6 orders of magnitude in body mass from egg to adult [27]. In basic size-based models, empirical relationships that link body mass, temperature and biological rates can be used to support parameterisation [28, 29]. More complex size-based approaches describe some of the differences between species in a size-structured community by incorporating information on traits. A species’ maximum (asymptotic) size is often treated as the main trait accounting for differences [30–33]. Once maximum size is assigned, life history theory can be used to estimate parameters such as size at maturity and reproductive output [31, 33]. More complex size- and species- based models incorporate some species-specific information directly, but use general size-based relationships to describe other components of the system [34–36]. The generality of size-based models also facilitates one- and two-way coupling with biogeochemical and low trophic level models [36–41].

Here, we develop a size-based equilibrium model that draws on established principles in macroecology, life history theory and food web ecology to predict the global abundance, production, distribution and size-structure of marine consumers and to assess the effects of parameter uncertainty on these predictions. Rates and magnitudes of energy flux from primary producers to consumers depend on primary production, transfer efficiency, predator and prey body mass and temperature. Resulting predictions of consumer size-structure and abundance are used to calibrate a more complex dynamic size and trait- based model [33], modified to account for the effects of temperature on biological rates [28]. In contrast with the size-based equilibrium model, the size and trait- based model enables the direct exploration of the effects of different rates of fishing on unexploited biomass. Outputs of the analysis with the size and trait- based model are estimates of unexploited biomass, production and size-structure as well as potential fisheries yield for different consumer size-classes by ecosystem and globally; given defined mortality rates and fisheries selectivity. Our results provide a baseline for assessing human impacts and predicting the contribution of marine animals to biogeochemical processes and are compared with the disparate results from existing models.

## Materials and Methods

Biomass, production and size-structure of consumers in any defined size range were calculated from the primary production available to support them, accounting for the factors that affect the rate and efficiency of energy processing. These factors are (i) temperature, which affects rates of metabolism and hence growth and mortality [28, 29], (ii) the size of phytoplankton and the predator to prey body mass ratio (PPMR), which determine the number of steps in a food chain [11], and (iii) trophic transfer efficiency (TE), a measure of energy conserved and lost at each step in the chain [32, 42]. When adopting this approach (hereafter the macroecological model) the PPMR is the realised PPMR (resulting from choices made by the predator given available prey) while in the size- and trait based model [33] PPMR is expressed as a preference (indicating choices made if available prey were equally abundant). Consequently, the preference PPMR in a size- and trait-based model (*μ*_*P*_) is set to ensure that the realised (output) PPMR is equal to the realised (input) PPMR (*μ*_*R*_) in the macroecological model. TE in the macroecological model was estimated directly, while, in the size- and trait-based model, it was an output resulting from the processes accounting for energy transfer; including the probability of encountering prey, the probability of prey capture and gross growth efficiency [32].

### Macroecological model

The macroecological model was used to estimate numbers (*N*) and biomass (*B*) of consumers at body mass (*M*), based on the assumption that the amount of energy transferred through a size-based food chain from phytoplankton to higher trophic levels depends on their size composition and abundance, *μ*_*R*_ and TE. Size composition of the phytoplankton community was estimated from primary production and temperature (*T*) using empirical relationships [43] and, in turn, size composition was used to estimate particle export ratios [44]. *N* and *B* were estimated in the water column from the surface to the euphotic depth (*Z*_*e*_), or the mixed layer depth if this was deeper. We made the simplifying assumption that all primary production occurred in this zone and was used in this zone and did not explicitly model communities, including detritivores, which live in deeper water. All model outputs were depth integrated and thus expressed per unit area.

#### Phytoplankton size structure

When smaller phytoplankton are more abundant the length of food chains increases (where “length” is defined as the number of prey to predator steps to reach a consumer of defined body mass and assuming *μ*_*R*_ does not depend on prey size) and carbon export from surface waters is lower [43, 44]. For these reasons the size-structure of the phytoplankton community that supports consumer production needs to be established.

The metrics used to describe phytoplankton structure were the (i) slope of the phytoplankton size spectrum, *b*_*P*_ (where the size-spectrum is the relationship between log_10_ *M* (pg C) and log_10_ *B* (pg C m^-3^)), (ii) intercept of the phytoplankton size spectrum, *a*_*P*_, (iii) *M*_*P*50_, the *M* of phytoplankton which, when individuals are ranked by *M*, corresponds to 50% of cumulative primary production, (iv) *M*_*P*1090_, log_10_ of the body mass range of phytoplankton which, when individuals are ranked by *M*, accounts for 10% to 90% (= 80%) of cumulative primary production. *M* at the lower and upper extremes of the phytoplankton size distribution, *M*_*P*0_ and *M*_*P*100_, as needed to determine the proportion of cells in different mass ranges, were calculated from *M*_*P*50_ and *M*_*P*1090_ owing to the variable tails of the real distributions [43]. Particle export ratio *ϕ*_*E*_, was estimated from the proportion of phytoplankton cells smaller than 5μm [44].

Slopes *b*_P_ and intercepts *a*_P_ of the phytoplankton size spectra were estimated from empirical relationships with primary production *P*_*P*_ using the approach and data of Barnes et al [43]

$$b_{P}=\alpha +\beta_{1}\;{\text{log}}_{10}\;P_{P}$$ (1)

$$a_{P}=\alpha +\beta_{1}\;{\text{log}}_{10}\;P_{P}$$ (2)

where *α* and *β*_1_ are fitted coefficients. *M*_*P*50_ and *M*_*P*1090_ were estimated from empirical relationships with *T*_*C*_ (°C) and primary production *P*_*P*_ (g C m^-2^ d^-1^)

$$M_{\text{P}50}=10^{\alpha +\beta_{1}\;{\text{log}}_{10}\;P_{P}+\beta_{2}T_{C}}$$ (3)

$$M_{P1090}=10^{\alpha +\beta_{1}\;{\text{log}}_{10}\;P_{P}+\beta_{2}T_{C}}$$ (4)

where *α*, *β*_1_ and *β*_2_ were the fitted coefficients. In simulations to address uncertainty, mean parameter values in Eqs 1–4 were replaced with values drawn from normal or multivariate normal distributions defined by the mean, standard deviation and covariance between parameters (Table 1).

**Table 1:** Predictors of phytoplankton community size structure. Means and standard deviations of the α, *β*_1_ and *β*_2_ coefficients used to predict the size structure of the phytoplankton community as a function of primary production and temperature [43].

Parameter	Coefficient					
	*α* (mean)	*α* (s.d.)	*β*_1_ (mean)	*β*_1_ (s.d.)	*β*_2_ (mean)	*β*_2_ (s.d.)
*b*_*P*_	-1.715	0.088	0.182	0.032	0	-
*a*_*P*_	8.087	0.258	0.554	0.093	0	-
*M*_*P*50_	-0.545	0.344	0.864	0.116	-0.078	0.006
*M*_*P*1090_	0.266	0.097	0	-	0.025	0.006

*M*_*P*0_ and *M*_*P*100_ were determined from *M*_*P*50_ and *M*_*P*1090_ by incorporating an additional 20% of integrated production and thus ensuring integrated *P*_*P*_ was equal to total *P*_*P*_,

$$M_{\text{P}0}={\left(-\frac{5}{4}M_{\text{P}50}^{b_{P}+1}+\frac{5}{4}M_{\text{P}10}^{b_{P}+1}+M_{\text{P}50}^{b_{P}+1}\right)}^{\frac{1}{b_{P}+1}}$$ (5)

$$M_{\text{P}100}={\left(\frac{5}{4}M_{\text{P}90}^{b_{P}+1}-\frac{5}{4}M_{\text{P}50}^{b_{P}+1}+M_{\text{P}50}^{b_{P}+1}\right)}^{\frac{1}{b_{P}+1}}$$ (6)

#### Estimating phytoplankton export production from cell size

The predicted size structure of the phytoplankton community (as described with *b*_*P*_, *a*_*P*_ *M*_*P*0_ and *M*_*P*100_) was used to estimate the fraction of production attributed to small cells *ϕ*_*S*_. Small cells were defined as cells of *M* ≤ *M*_*ref*_ where *M*_*ref*_ was set at 5μm Equivalent Spherical Diameter (12.9 pg C). *ϕ*_*S*_ was calculated from the integrated phytoplankton production from *M*_*ref*_ to *M*_*P*100_ as a fraction of the integrated production from *M*_*P*0_ and *M*_*P*100_.

$$\begin{array}{l} \phi_{S}=\;(\frac{1}{2}b_{P}\;{\text{log}}_{10}\;M_{ref}{}^{2}+a_{P}\;{\text{log}}_{10}\;M_{ref}-\frac{1}{2}b_{P}\;{\text{log}}_{10}\;M_{P0}{}^{2}-a_{P}\;{\text{log}}_{10}\;M_{P0})/\\ \left(\frac{1}{2}b_{P}\;{\text{log}}_{10}\;M_{P100}{}^{2}+a_{P}\;{\text{log}}_{10}\;M_{P100}-\frac{1}{2}b_{P}\;{\text{log}}_{10}\;M_{P0}{}^{2}-a_{P}\;{\text{log}}_{10}\;M_{P0}\right) \end{array}$$ (7)

The proportion of large cells *ϕ*_*L*_ was defined as 1 − *ϕ*_*S*_. Estimates of *ϕ*_*S*_ and *ϕ*_*L*_ were used to estimate the fraction exported *ϕ*_*E*_ at any defined depth following Dunne et al [44]

$$\phi_{E}=(e^{T_{C}\times k_{D}}(\phi_{SD}\phi_{S}+\phi_{LD}\phi_{L})+Rr_{B}\phi_{L})/(1+R)$$ (8)

Where *k*_*D*_ is the temperature dependence of primary production, *ϕ*_*SD*_ is the proportion of small phytoplankton grazing going to detritus referenced to 0°C, *ϕ*_*LD*_ is the proportion of large phytoplankton grazing going to detritus referenced to 0°C and *r*_*B*_ is the fraction of production leading to detritus protected by mineral. *R*, the vertically integrated remineralisation coefficient is estimated as

R=ZD/S (9)

Where *Z* is depth (m), *D* is the detritus removal rate constant (d^-1^) referenced to 0°C and *S* is the sinking rate of detritus (m d^-1^; [44]).

#### Proportion of primary production supporting primary consumer production

The proportion of primary production available to support production of primary consumers was estimated as the product of an assumed TE from primary producers to primary consumers *ε*_*P*_ (assumed to represent the product of assimilation efficiency and gross growth efficiency) modified with a multiplier for the relative export fraction *τ*_*R*_ to account for other losses of energy from the modelled system. Estimates of *ϕ*_*E*_ at the depth of analysis (*φ*_*EZ*_), as well as a reference depth (*ϕ*_*Eref*_) of 200m, were used to calculate *τ*_*R*_

$$\tau_{R}=(1-\phi_{EZ})-(1-\phi_{Eref})+1$$ (10)

*τ*_*R*_ modified the mean value of *ε*_*P*_ [8] to account for the observed increase in overall trophic transfer efficiency in food webs *ε* with depth [16]. We therefore assume that the effects of depth on *ε* all result from differences in *ϕ*_*E*_ and their effect on *ε*_*P*_. In simulations to address uncertainty, means and standard deviations of *ε*_*P*_ were set to recover the approximate distribution of reported values of TE [8], but we did not independently vary the parameters of the equations used to estimate *τ*_*R*_.

#### Consumer production and biomass

In the model, the relationship between primary consumer production and consumer production at any higher trophic level was determined solely by the transfer efficiency [3]. Production at a given body mass or trophic level was converted to biomass and numbers at the same body mass or trophic level based on the assumption that body size and temperature determined individual rates of production [28].

Production of an individual consumer *P*_*CI*_ was estimated from consumer *M* (*M*_*CI*_) and *T* [28, 29] using

$$P_{CI}=e^{\left(c_{1}-E/kT_{K}\right)}M_{CI}{}^{r}$$ (11)

where *c*_1_ is a fitted constant, *E* is the “activation energy of metabolism”, *k* is Boltzmann’s constant, *T*_*K*_ is temperature in Kelvin (°C+273) and *r* is the scaling of rate with *M*_*CI*_. Dividing by *M*_*CI*_, it follows that the mass specific production to body mass ratio (*P*_*CI*_ / *M*_*CI*_) will be

$$P_{CI}/M_{CI}=e^{\left(c-E/kT\right)}M_{CI}{}^{r-1}$$ (12)

The ratio *P*_*CI*_ / *M*_*CI*_ was used to convert consumer production *P*_*C*_ in any defined body mass class to biomass (*B*_*C*_ = (*P*_*CI*_ / *M*_*CI*_)×*P*_*C*_). Numbers *N*_*C*_ in the same body mass class were estimated as *N*_*C*_ = *B*_*C*_ / *M*_*CI*_. In simulations to address uncertainty, mean values of parameters for estimating the mass specific production to body mass ratio from body mass and temperature were replaced with distributions defined by the mean and standard deviation from fits to data [28, 29].

Biomass size-spectrum

Slope of the consumer biomass size-spectrum (*b*_*C*_; relationship between log_10_ *M*_*CI*_ and log_10_ *B*_*CI*_) was estimated as [42, 45, 46]

$$b_{C}=M_{C}{}^{({\text{log}}_{10}\;\varepsilon_{S}/{\text{log}}_{10}\;\mu_{S})+b_{S}}$$ (13)

Where *μ*_*S*_ is the predator to prey mass ratio (log_10_) for secondary consumers, *ε*_*S*_ the trophic transfer efficiency for secondary consumers expressed as a proportion and *b*_*S*_ is the theoretical slope of the biomass size spectrum when energy is shared between individuals [45].

While *b*_*C*_ is unlikely to change with primary production [47, 48] the intercept of the consumer size-spectrum *a*_*C*_ has to be established to estimate *P*_*C*_ and hence *B*_*C*_ at *M*_*C*_. The B for primary consumers of mass log_10_ *M*_*P*50_ + *μ*_*R*_, where *μ*_*R*_ is the realised log_10_ predator-prey mass ratio for primary consumers, was estimated from *ε*_*P*_, *P*_*P*_ and *τ*_*R*_ as

$$B=\varepsilon_{P}P_{P}\tau_{R}$$ (14)

thus

$$a_{c}=({\text{log}}_{10}\;B)-(b_{c}({\text{log}}_{10}\;M_{P50}+\mu_{P}))$$ (15)

#### Implementation

The entire size-spectrum was discretised into *M* units of 0.1 (log_10_) for analysis. *N* and *B* were expressed per unit area of sea surface. In runs of the macroecological model (10000 per grid cell), parameters were drawn randomly from normal distributions with mean and standard deviation based on fits to data (Table 2). When parameters in the same equation were correlated, we drew randomly from multivariate normal distributions. We also assumed weak covariance between *μ*_*R*_ and *ε*_*P*_ [48], but recognise that available data lead to different conclusions about the extent of this covariance (see discussion). Conversions from primary consumer carbon to *M* (wet weight) assumed *C*(*g*) = 0.32*M* (*dry*, *g*) and *M* (*dry*, *g*) = 0.11*M* (*g*) [49]. Model results were expressed as medians and percentiles calculated from the distribution of output values. The model was implemented in Core R [50] with additions from MASS [51] and ggplot2 [52]. Model parameters are summarised in Tables 1 and 2.

**Table 2:** Values and sources of parameters in the macroecological model. When means and standard deviations are presented these were used to define the distributions used in the simulations. If no standard deviation is presented the value of the parameter was assumed to be fixed.

Parameter	Description	mean	s.d.	Source
*ε*_*P*_	log_10_ base trophic transfer efficiency to primary consumers (before additional losses)	-0.665	0.087	calibrated to [8]
*ε*_*S*_	log_10_ trophic transfer efficiency to secondary consumers	-0.936	0.170	[8]
*μ*_*P*_	log_10_ realised predator-prey mass ratio for primary consumers	3	0.35	[53], for copepods
*μ*_*S*_	log_10_ realised predator-prey mass ratio for secondary consumers	3	0.72	[25], median
*R*	scaling of individual production with body mass	0.755	0.003	[28]
*ϕ*_*LD*_	proportion of large phytoplankton grazing going to detritus and referenced to 0°C	0.74	-	[44]
*ϕ*_*SD*_	proportion of small phytoplankton grazing going to detritus and referenced to 0°C	0.14	-	[44]
*c*_1_	constant in equation linking individual production and body mass	25.22	-	[28]
*D*	detritus removal rate constant	0.4	-	[44]
*E*	activation energy of metabolism (eV)	0.6	-	[28]
*k*	Boltzmann’s constant (eV Kelvin^-1^)	8.62×10^−5^	-	[28]
*k*_*D*_	temperature dependence of primary production (°C^-1^)	-0.032	-	[44]
*r*_*B*_	proportion of production that leads to detritus protected by mineral	0.0228	-	[44]
*S*	sinking rate of detritus (m d^-1^)	100	-	[44]

### Size- and trait-based model

#### Background and assumptions

The dynamic size and trait-based model of Andersen and Beyer [30] mimics a food web comprising a range of pseudo-species (hereafter “species”) defined by asymptotic body sizes and can be parameterised to span the range of asymptotic body sizes encountered in ecosystems (e.g. [54]). The main model assumptions are (i) the consumption *C* of an individual of mass *M* is proportional to *M* ^*n*^ and (ii) individuals eat smaller individuals with a preferred PPMR(*μ*_*P*_) and a volumetric search rate proportional to *M* ^*q*^ [30, 33]. These assumptions are used to derive the community size spectrum. If the size-spectrum is in equilibrium every individual encounters sufficient food to meet its required consumption [30, 33].

The original dynamic size and trait-based model [30], was subsequently modified by Hartvig et al. [33] and the code has been translated from Matlab to R and subsequently published [55]. Here, the model was further modified to include the effects of varying primary production and temperature on abundance and rates. With these modifications the model is well suited to global analyses because it generalises food web processes with size-based predator-prey interactions that lead to growth and mortality and can be used to assess the effects of additional mortality (in this case fishing) on the food web.

#### Model setup and modifications

Consumers were assumed to vary in size from 0.001 g to the asymptotic mass (*M*_∞_) of the largest species (10^6^ g). Thirteen species were included in the model with *M*_∞_ of 1 to 10^6^ g and *M*_∞_ was evenly distributed in log space. Modelled species were supported by a resource spectrum of *M* from 1^−10^ to 0.1 g. Collectively, the resource and consumer spectra were discretised into 150 *M* classes evenly distributed in log space.

To modify ecological rates defined at a reference temperature *T*_*Kref*_ to rates at a local temperature *T*_*K*_ we used the multiplier *τ*_*T*_

$$\tau_{T}=e^{(-E/k){\left(1/T_{K}-1/T_{Kref}\right)}_{}}$$ (16)

A temperature of 283 K was adopted as *T*_*Kref*_. Non predation natural mortality (denoted as *μ*_0_ in [33]), the pre-factor for standard metabolism (*k* in [33]) and maximum food intake rate (*h* in [33]) were all multiplied by *τ*_*T*_. Consequently the ratio between food intake and standard metabolism at the individual level was not affected by temperature, but the higher rate of food intake at higher *T*_*K*_ led to higher prey mortality. All scaling exponents for relationships between rates and *M* were assumed to be unaffected by *T*_*K*_. Reproductive output from the species was reintroduced to the consumer spectrum at 0.005 g [33].

*N* and *B* in any mass increment (*M*, *M* + *dM*) were expressed per unit volume, and converted to *N* and *B* per unit area based on the simplifying assumption that the depth limit of the productive volume was the deeper of the euphotic or mixed layer depth. Euphotic depth was estimated from chlorophyll concentration [56].

To convert between area and depth-integrated *N* and *B* we modelled production within the euphotic depth, *Z*_*e*_, or the mixed layer depth when this was deeper. *Z*_*e*_ was estimated from chlorophyll concentration (mg m^-2^) with a relationship for depths to 180m [56]; *Z*_*e*_ was otherwise constrained to 180m. This approach was also used to estimate *Z*_*e*_ for runs of the macroecological model.

$${\text{log}}_{10}\;Z_{e}=2.1236+0.9325{\text{log}}_{10}\;Chl-1.4264{\text{log}}_{10}\;Chl^{2}+0.5278{\text{log}}_{10}\;Chl^{3}-0.0762{\text{log}}_{10}\;Chl^{4}$$ (17)

#### Calibration and implementation

The dynamics of the resource spectrum [33] depend on a carrying capacity (*κ*) and regeneration rate (*r*_0_) [33] and constrain the total abundance of consumers. These parameters were calibrated by minimising differences in *b*_*C*_, as predicted with the macroecological and trait-based models, for consumers of body mass 10^2^ to 10^4^ g. For the trait-based model, *b*_*C*_ was taken as an annual mean over 60 years, after running the model for a minimum of 120 years to achieve a relatively stable equilibrium size distribution (longer periods were occasionally required at low temperatures and with low primary production). The calibration provides a method to account for the effects of lower trophic level processes (Eqs 1–10) on relative production and biomass of consumers in different locations. Following calibration of parameters the median *b*_*C*_ values from the two models at F = 0 were within ± 2% of each other during subsequent runs and the 25th and 75th percentile values of *b*_*C*_ were within ± 5%. The two exceptions were LME 26 (Mediterranean Sea) and FAO area 37 (Mediterranean and Black Sea) where no stability was achieved in *b*_*C*_ estimates from the size- and trait-based model. These are regions where the GCM predicts that primary production is lower than the primary production estimated from remote sensing and other sources of data. Exploratory analyses demonstrated that stability was achieved if primary production was increased. However, we chose to model all regions with the same GCM inputs in this analysis, rather than make adjustments based on regional information. We did not conduct a full analysis of uncertainty with the size and trait-based model owing to the need to tune parameters and the absence of sufficient estimates to obtain probability distributions for most model parameters, but calibrations for the 25^th^, 50^th^ and 75^th^ percentiles of *b*_*C*_ at *F* = 0 were used to assess the consequences of uncertainty in assumptions about unexploited community biomass.

The core calculations for the size and trait-based model [33] were implemented with the R package mizer [55]. In addition to modifying functions to introduce temperature effects and obtain required outputs, changes from previous parameterisations [33] were made to ensure persistence of all species when the model was run without fishing and to give plausible rates and trajectories of individual and population growth for all observed combinations of temperature and primary production [33] (S1 Table).

#### Environmental forcing

The environmental data used to force the macroecological and size- and trait-based models comprised annual mean estimates of depth integrated primary production (g C m^-2^ d^-1^), chlorophyll (mg Chl m^-2^) and sea surface temperature (°C), which were derived from monthly predictions for the years 2010, 2011 and 2012. Chlorophyll and primary production were obtained from the Mercator Ocean Project [57] (Global Biogeochemical Analysis Product, BIOMER1V1 monthly 0.5° degree resolution) and monthly temperature data from the Mercator Ocean physical NEMO model (PSY3V3R1) at 0.25° degree resolution. Inputs to the size-based models were allocated to a 0.5° grid, which covered the GCM domain and cells assigned a mixed layer depth (m) [58], total depth (m) [59] and sea surface area (km^2^) as well as being assigned to LME [60] and FAO areas outside LME [60, 61].

#### Assessing fisheries impacts

The effects of fishing were investigated with the size and trait-based model [33, 53]. Selectivity was defined by species and size. Mortality rates for individual species were scaled in relation to *F*_35_, the rate of fishing mortality estimated to reduce spawning stock biomass (SSB) per recruit to 35% of the SSB per recruit expected in the absence of fishing, which was initially estimated from *M*_∞_ and an assumed *M* at knife-edge selection [62]. The selectivity scenarios considered (Table 3) were (A) the same selectivity for species and sizes in LME and FAO areas (to support comparison of relative fisheries potential and impacts between areas) with the minimum size of any individual targeted set at 8 cm, (B) as ‘A’ with the minimum size of an individual targeted increased to 20 cm in the FAO areas only, (C) minimum size of an individual targeted set at 7 cm in LME and 20 cm in FAO areas (to mimic industrial fisheries, which may exist or may be developed in some LME), with individuals larger than 48 cm of any species targeted and subject to the same *F* (to mimic fisheries where the capacity to control size at selection for larger species is weak), and (D) the same selectivity for species and sizes in LME and FAO areas (to support comparison of relative fisheries potential and impacts among areas) with the minimum size of any individual targeted set at 20cm. When investigating community-wide effects of fishing, relative *F*’s for all species was scaled by the same multiplier (0 to 3 in steps of 0.05) and local *T* correction.

**Table 3:** Assumed selectivities and fishing mortality rates for modeled species. Different selectivities, fishing mortality rates (*F*) and relative fishing mortality rates between species (*F*_*rel*_) were assumed in the four scenarios. Asymptotic length (*L*_*∞*_) in cm was estimated from *M*_∞_ in g assuming *M* = 0.01*L* ^3^ and length at first capture (*L*_*c*_) was based on an assumed age at first capture [62]. When values are shown in bold they apply only to areas inside LME and not to the FAO areas.

	Scenario A			Scenario B			Scenario C			Scenario D		
*L*_∞_	*L*_*c*_	*F*	*F*_*rel*_	*L*_*c*_	*F*	*F*_*rel*_	*L*_*c*_	*F*	*F*_*rel*_	*L*_*c*_	*F*	*F*_*rel*_
5	-	-	-	-	-	-	-	-	-	-	-	-
7	-	-	-	-	-	-	-	-	-	-	-	-
10	-	-	-	-	-	-	-	-	-	-	-	-
15	-	-	-	-	-	-	**7**	**0.6**	**1.00**	**-**	**-**	**-**
22	8	0.43	0.88	**8**	**0.43**	**0.88**	**8**	**0.6**	**1.00**	**-**	**-**	**-**
32	16	0.49	1.00	**16**	**0.49**	**1.00**	**16**	**0.49**	**0.82**	**-**	**-**	**-**
46	20	0.3	0.61	20	0.3	0.61	20	0.3	0.50	20	0.3	1.00
68	28	0.19	0.39	28	0.19	0.39	28	0.19	0.32	28	0.19	0.63
100	38	0.16	0.33	38	0.16	0.33	38	0.13	0.22	38	0.16	0.53
147	60	0.12	0.24	60	0.12	0.24	48	0.13	0.22	60	0.12	0.40
215	85	0.08	0.16	85	0.08	0.16	48	0.13	0.22	85	0.08	0.27
316	108	0.06	0.12	108	0.06	0.12	48	0.13	0.22	108	0.06	0.20
464	127	0.04	0.08	127	0.04	0.08	48	0.13	0.22	127	0.04	0.13

## Results

### Overview

Median consumer biomass of 4.9 billion tonnes was predicted for the body mass range from 1g to 1000kg. Accounting for parameter uncertainty led to wide uncertainty intervals, with 90% of simulations between 0.3 and 26.1 billion tonnes. Most uncertainty was driven by uncertainty in TE. For the consumer size range of 100 g to 10 kg, which is usually dominated by fish and squids, the macroecological model predicted median biomass of 1.6 billion tonnes, with 50% uncertainty intervals of 0.6 to 3.5 billion tonnes. Predicted maximum multispecies sustainable yield (MMSY) for medium-sized (maximum body mass 1–10 kg) and large species (> 10 kg), as predicted with the size- and trait-based model, ranged from 50 to 65 million tonnes yr^-1^ and 19 to 26 million tonnes yr^-1^ respectively, depending on gear selectivity. Predicted MMSY for fishes of all sizes depended primarily on the extent to which small fishes were selected. Targeting individuals < 20 cm from species of low maximum mass <1kg led to a ≈4 fold increase in predicted MMSY (assuming the median estimate of unexploited biomass), from 130 to 512 million tonnes yr-1. Total depletion of all consumers at MMSY (relative to the unfished state) is predicted to be less than 50% if small consumers (here taken as individuals of <20 cm length from species with a maximum mass of 1 kg) were not targeted. However, this belies much larger reductions, of 80% or more, in the relative biomass of the largest modelled species (>10 kg) at overall MMSY because their *F*_MMSY_ is lower than *F*_MMSY_ for the modelled community as a whole.

### Macroecological model

**Fig 1:**
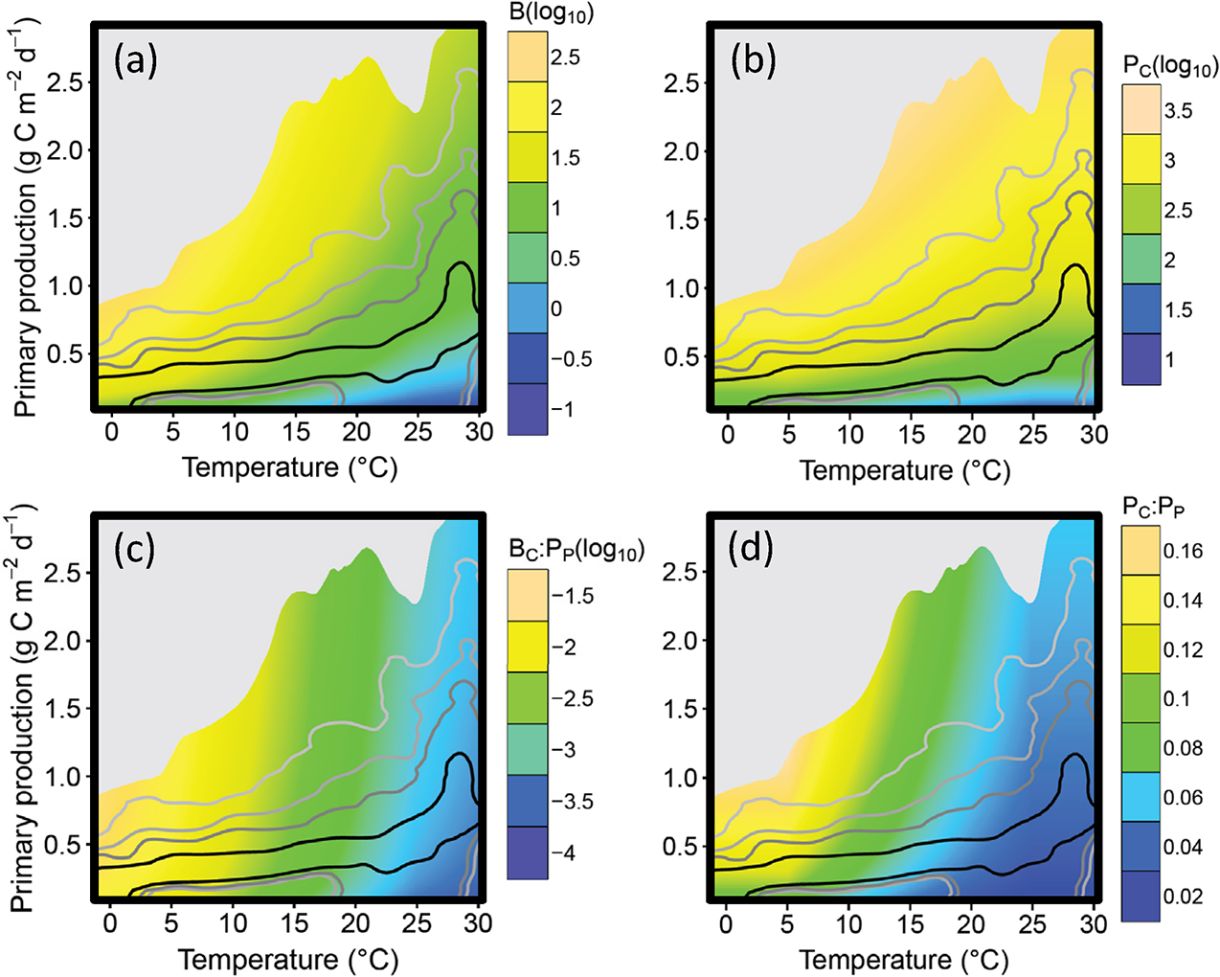
Predicted effects of temperature on production and biomass. Modelled relationships between temperature *T*_*C*_ and daily primary production *P*_*P*_ and (a) median estimated consumer biomass *B* (g m^-2^ in *M* range 1 to 10^6^ g), (b) median estimated consumer production *P*_*C*_ (g m^-2^ in *M* range 1 to 10^6^ g), (c) ratio of primary production (g m^-2^ yr^-1^) to consumer biomass *B*_*C*_ (g m^-2^ yr^-1^ in *M* range 1 to 10^6^ g) and (d) ratio of primary production (g m^-2^ yr^-1^) to consumer production *P*_*C*_ (g m^-2^ yr^-1^ in *M* range 1 to 10^6^ g). In all simulations *Z*_*e*_ was assumed to be fixed at 50 m and *Z* at 200 m. Chlorophyll concentration was estimated from primary production using a relationship established from the GCM outputs. Values of *T*_*C*_ and *P*_*P*_ that fell outside ranges including 99.99% of GCM outputs for the world’s oceans are masked. Contours indicate combinations of *T*_*C*_ and *P*_*P*_ that include 70% (black), 90%, 95% and 99% (pale grey) of GCM outputs.

Median predictions of the macroecological model for consumers of 1g to 1000kg body mass and with *T* and *P*_*P*_ ranges spanning the range of inputs to the global simulations suggest that consumer production is broadly correlated with primary production but *P*_*C*_ increases faster with *P*_*P*_ at lower temperatures (Fig 1Á). The decrease in consumer biomass with temperature at a given *P*_*P*_ is slower than the rate of decrease in *P*_*C*_ (Fig 1B). Consumer biomass and production per unit primary production are greater at lower temperatures and with high primary production (Fig 1C and 1D). The ratios are lowest at high temperatures and when primary production is low, but the range of primary production values predicted in GCM output at high temperatures is narrow and will have a small effect on the ratio. A given change in primary production was predicted to have a much greater effect on consumer production at lower temperatures (Fig 1D). These relationships are consistent with those for consumers in the narrower body mass range of 100g to 10kg (S1 Fig).

**Fig 2:**
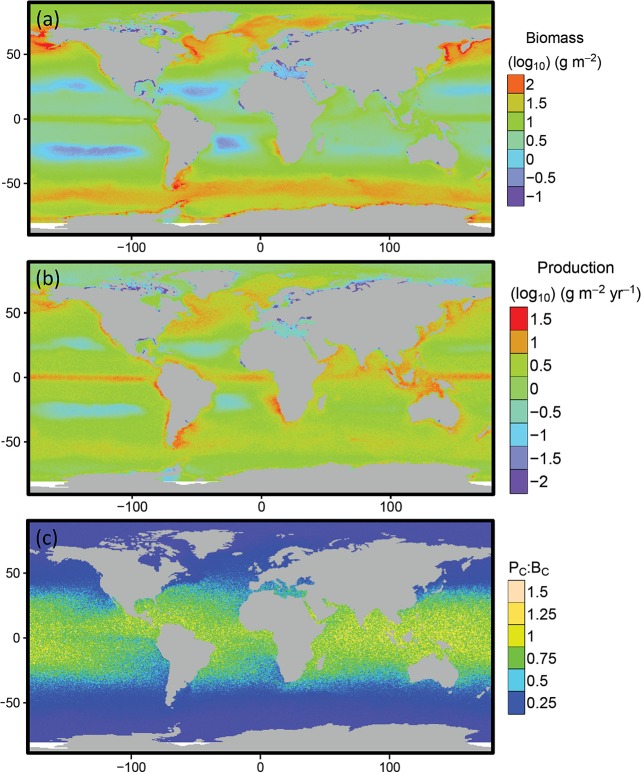
Predicted spatial distributions of consumer biomass and production. Predicted global distribution of (a) consumer biomass, (b) production and (c) the consumer production: biomass ratio for individuals of body mass 1 to 10^6^ g. Areas in white, predominantly in the southern ocean, are marine areas not included in the GCM domain.

**Fig 3:**
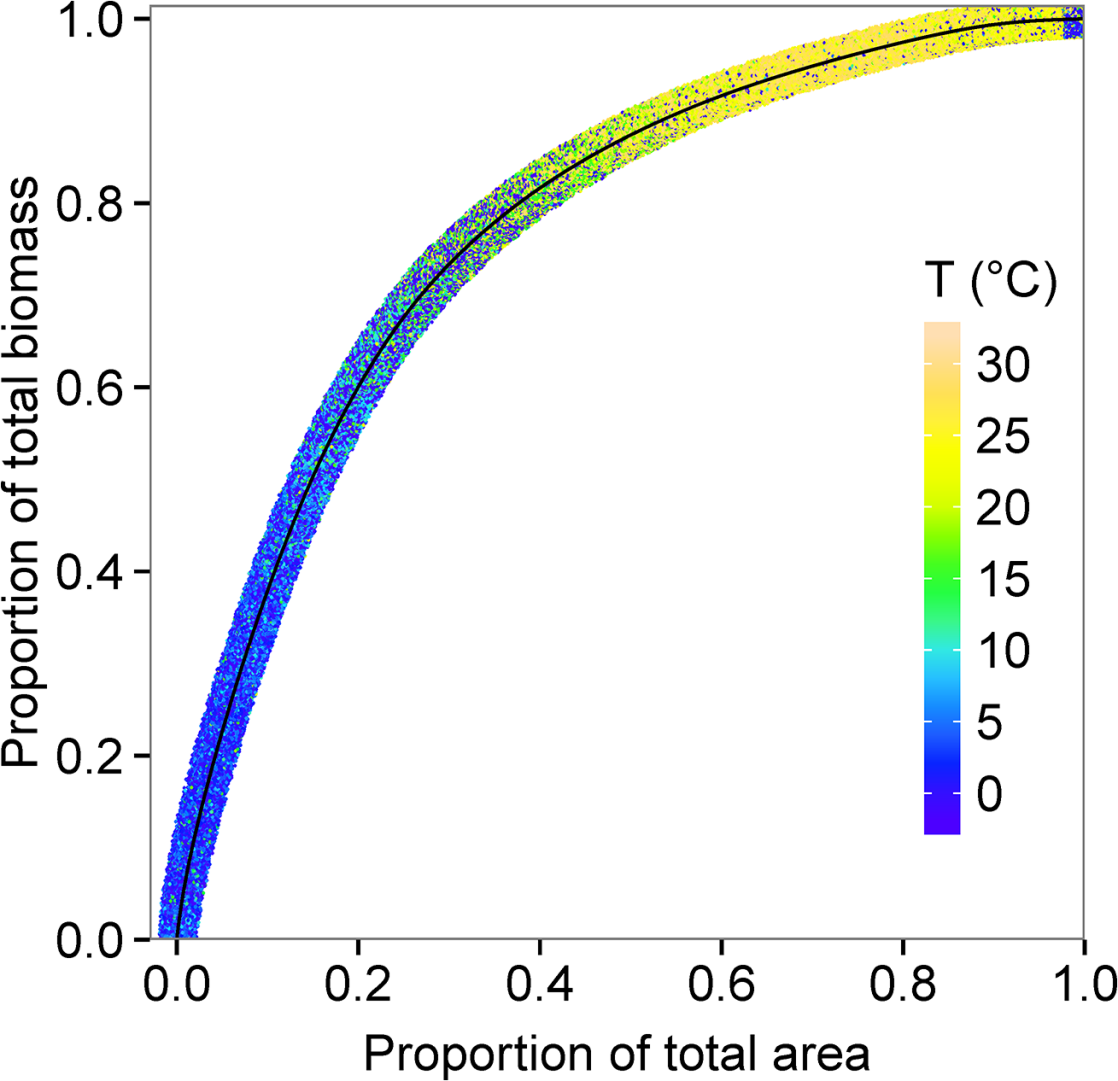
Predicted relationship between cumulative biomass of consumers and ocean area. The colour scale (extended vertically for visibility) indicates sea surface temperatures in 0.5° grid cells contributing biomass values at each point on the cumulative relationship (black line).

Median consumer biomass (g m^-2^) for individuals of 1 g to 10^6^ g body mass is predicted to be highest in mid to high latitudes (45° to 80° N and S) and especially low in the Atlantic and Pacific gyres and central Mediterranean (Fig 2A, S2 Fig). There are also areas with low predicted biomass close to some Atlantic and Arctic coasts. Relatively high biomass is predicted in the equatorial upwelling and upwellings on the western coasts of North and South America and southern Africa (Fig 2A, S2 Fig). Eighty percent of consumer biomass is predicted to be distributed in less than one third of the total area of the oceans, in predominantly cooler regions (Fig 3). Consumer production is highest in a circumglobal band around the equator, in other upwellings in low and mid latitudes, the North Pacific and in the south Atlantic Ocean east of Argentina (Fig 2B, S2 Fig). Production is relatively low in the Atlantic and Pacific gyres. Production to biomass ratios of consumers peak in a circumglobal band from 30° N to 30° S (Fig 2C).

**Fig 4:**
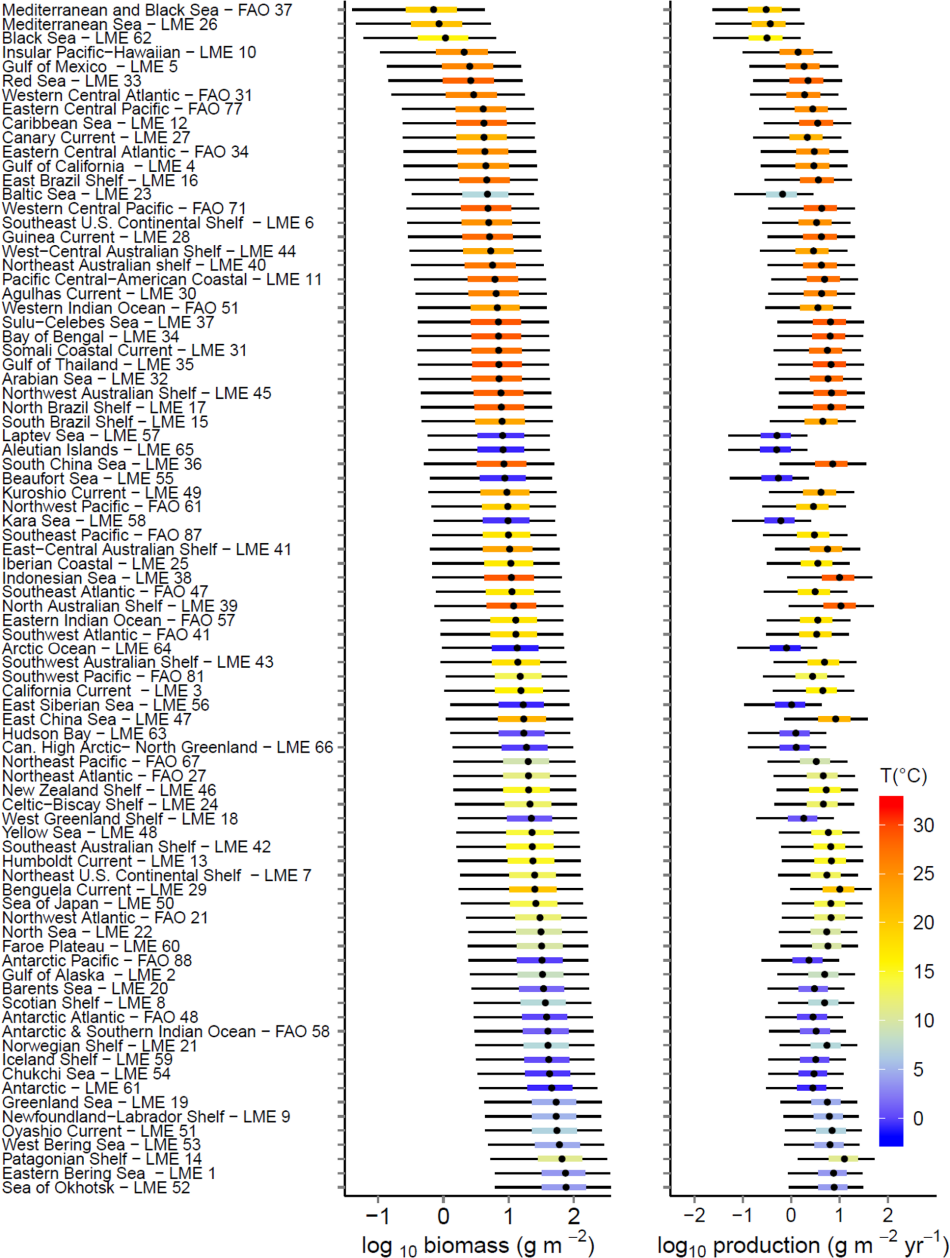
Predicted consumer biomass and production by location. Estimated biomass and production of consumers of 1 to 10^6^ g body mass by LME and FAO area as predicted with the macroecological model. Points represent median biomass estimates, coloured bars (mapped to sea surface temperature) the 25^th^ to 75^th^ percentiles and black lines the 5^th^ to 95^th^ percentiles. For corresponding absolute estimates of biomass and production see S3 Fig and for further details of the LME see S2 Table. Uncertainty intervals show the effects of parameter uncertainty in the macroecological model but do not account for structural uncertainty or uncertainty in primary production and temperature inputs.

The 90% uncertainty intervals for biomass and production predictions within LME and FA0 areas (per unit area values in Fig 4, absolute values in S3 Fig, details of LME and FAO areas in S2 Table) span around 1.5 orders of magnitude (≈30 fold) and 50% uncertainty intervals (25^th^ to 75^th^ percentiles of biomass and production estimates) span ≈5 fold. For all areas combined, the median estimated biomass of consumers with no fishing in body mass classes 1g to 1000kg is 4.9 × 10^9^ tonnes with just over 1.6 × 10^9^ tonnes in the size classes 100g to 10kg, which are consistently dominated by fishes and squids (Table 4). Uncertainty in TE and PPMR accounted for >80% of the uncertainty in biomass estimates (based on simulations with TE and PPMR fixed at mean values).

**Table 4:** Uncertainty in predictions of global consumer biomass and production. Consumer biomass and production estimates and associated uncertainties for individuals of 10^1^ to 10^6^ g body mass and 10^2^ to 10^4^ g body mass as predicted with the macroecological model. Estimates of the median, 25^th^ and 75^th^ percentiles for biomass of consumers of 10^2^ to 10^4^ g body mass by LME or FAO area were used to tune the size and trait-based model.

Percentile	10^0^ to 10^6^ g body mass				10^2^ to 10^4^ g body mass			
	Biomass (10^9^ t)	Biomass (g m^-2^)	Production (10^9^ t yr^-1^)	Production (g m^-2^ yr^-1^)	Biomass (10^9^ t)	Biomass (g m^-2^)	Production (10^9^ t yr^-1^)	Production (g m^-2^ yr^-1^)
**5** ^**th**^	0.34	0.94	0.10	0.28	0.09	0.24	0.01	0.04
**25** ^**th**^	1.97	5.43	0.51	1.42	0.63	1.72	0.09	0.26
**50** ^**th**^	4.88	13.45	1.17	3.22	1.61	4.44	0.25	0.68
**75** ^**th**^	10.37	28.55	2.32	6.39	3.49	9.61	0.54	1.49
**95** ^**th**^	26.12	71.90	5.44	14.99	8.89	24.48	1.40	3.86

To compare our results with another recent estimate of marine consumer biomass [13], we also predicted biomass for consumers of 10^−5^ to 10^6^ g body mass. The median global biomass estimate was 1.4 × 10^10^ tonnes with 25^th^ and 75^th^ percentiles of 7.7 × 10^9^ and 2.3 × 10^10^ tonnes and 5^th^ and 95^th^ percentiles of 2.9 × 10^9^ and 4.6 × 10^10^ tonnes. The highest mean biomass value per unit area in LME or FAO areas (> 180 g m^-2^) was predicted in the East and West Bering Seas, Patagonian Shelf and Sea of Okhotsk

### Size- and trait-based model

**Fig 5:**
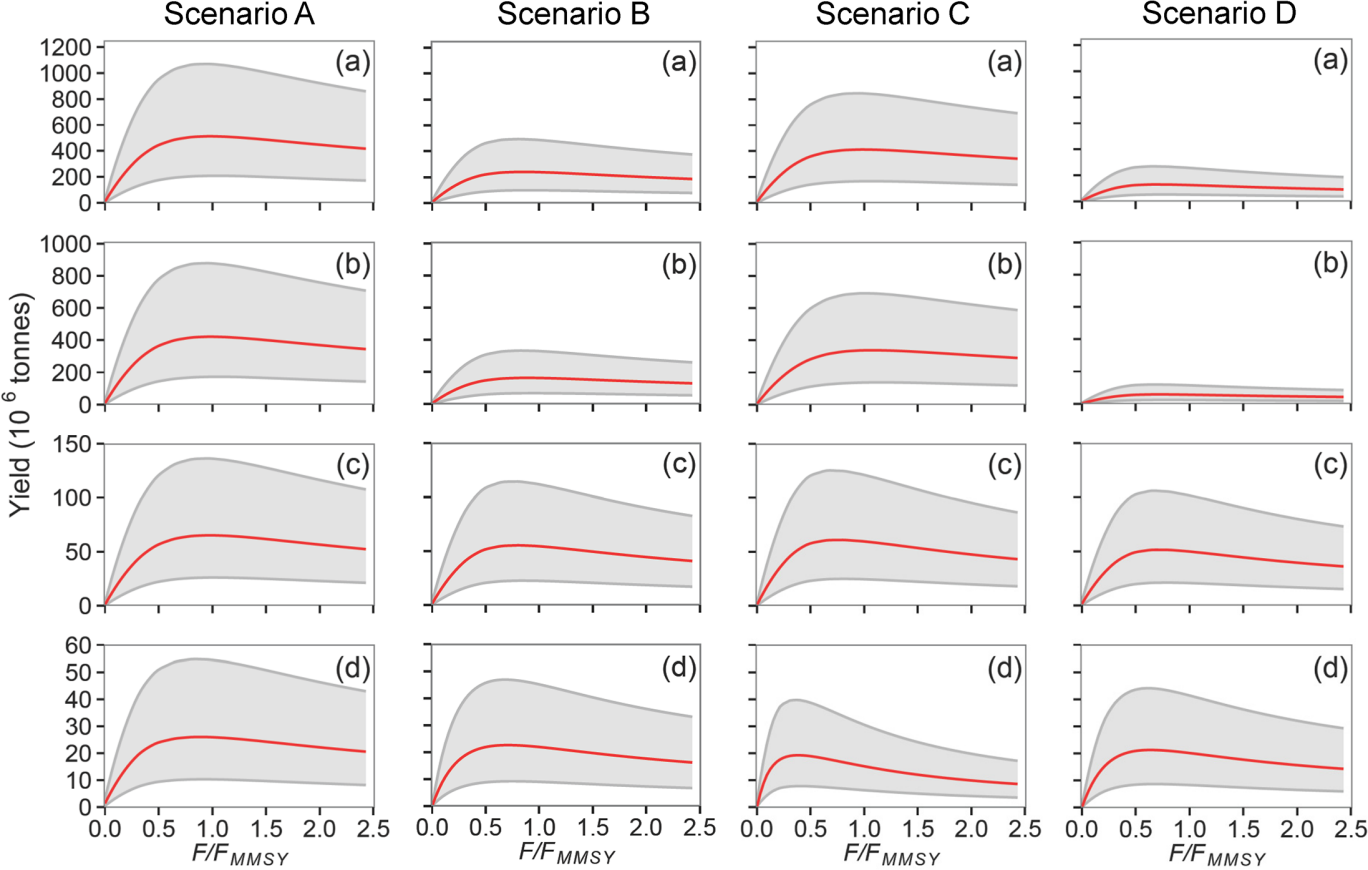
Predicted relationships between global yield and fishing mortality. Modelled changes in global yield as a function of *F* (expressed as a proportion of *F*_*MMSY*_ for all consumers of body mass > 100g) and selectivity. Each column presents results for one selectivity scenario (A to D, Table 3) and panels show yields by consumer for (a) all individuals of any body mass, (b) body mass < 1 kg (small), (c) 1 kg to 10 kg (medium) and (d) > 10 kg (large).

**Fig 6:**
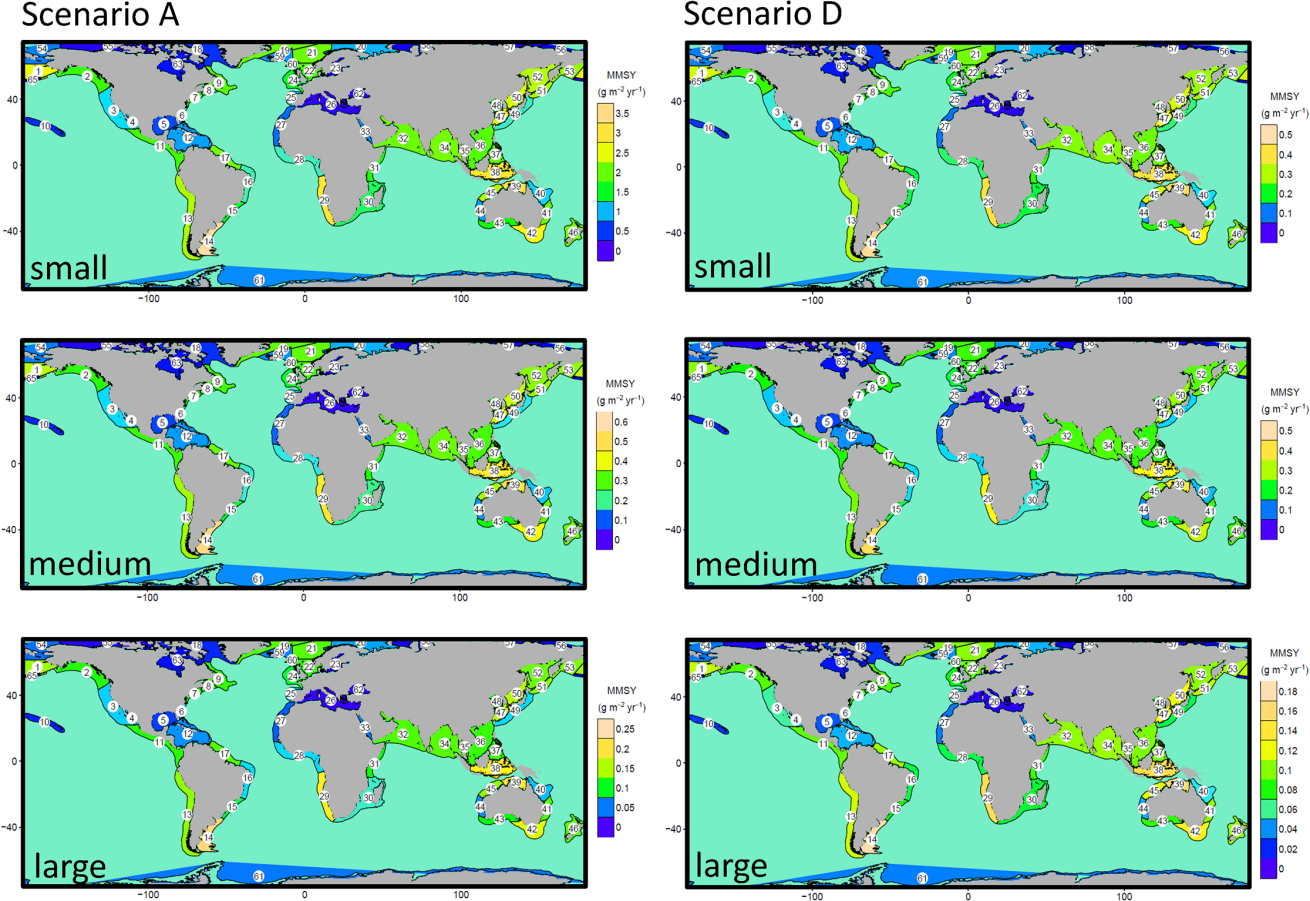
Predicted maximum multispecies sustainable yield in large marine ecosystems. Median estimate of the predicted maximum multispecies sustainable yield mapped by LME when fishing with selectivity Scenario A (or B) and D. Upper panel for small species (body mass <10^3^g), centre panel for medium-sized species (10^3^−10^4^ g) and lower panel for large species (>10^4^ g). For corresponding figures based on selectivity scenario C see S5 Fig.

Potential yields and the responses of the modelled community to fishing varied substantially with body size and the rates of fishing mortality and selectivity assumed (Table 3). Median global MMSY for consumers in all body size classes ranged from 130 to 512 million tonnes depending on the selectivity scenario assumed (Fig 5, Table 5). Predicted MMSY for medium-sized consumers (species with maximum body mass 1–10 kg) and large consumers (> 10 kg) was much lower and more stable in response to the changing selectivity patterns than total MMSY. Total MMSY was therefore dominated by yields of small individuals and when smaller individuals were selected the model predicted much higher global MMSY. When size-selection was assumed to be the same in LME and FAO areas (Table 3, scenarios A and D) the targeting of fishes < 20 cm (scenario A) led to a ≈4 fold increase in MMSY (Fig 5). When separable control of mortality of large species and individuals is not assumed (scenario C), MMSY for large consumers is attained at an *F* that is less than half that leading to MMSY for all consumers (Fig 5, scenario C, panel d). Differences in MMSY for small consumers (<1 kg) drove most of the variation in predicted total yield, and these differences were heavily influenced by the assumed selectivity scenario (Fig 5, for results by LME with scenarios A, B and D see Fig 6, for scenario C see S4 Fig). Potential yields of small consumers (species of maximum length < 1kg) were always <10 fold greater than that of large consumers in the scenarios A-C where individuals < 20cm are targeted (Fig 5) and drove the estimates of total MMSY (S5 Fig). Uncertainty in yield at *F*, based on the 25^th^ and 75^th^ percentiles for unexploited biomass, is typically ≈5 fold, but differences in yield resulting from differences in selection usually exceed this.

**Table 5:** Predicted effects of selectivity on maximum multispecies sustainable yield. Maximum multispecies sustainable yields for the four selectivity scenarios, by body mass range. Percentiles refer to the percentiles of unexploited biomass from the macroecological model that were used for calibrating the size- and trait-based model.

	Scenario A			Scenario B			Scenario C			Scenario D		
Body mass	25^th^	50^th^	75^th^	25^th^	50^th^	75^th^	25^th^	50^th^	75^th^	25^th^	50^th^	75^th^
**All**	208	512	1068	99	240	492	168	412	844	54	130	268
**< 10** ^**3**^ **g**	172	421	877	67	163	332	138	338	692	24	58	119
**10** ^**3**^ **-10** ^**4**^ **g**	26	65	136	23	55	114	25	61	125	21	51	106
**>10** ^**4**^ **g**	10	26	55	9	23	47	8	19	40	9	21	44

**Fig 7:**
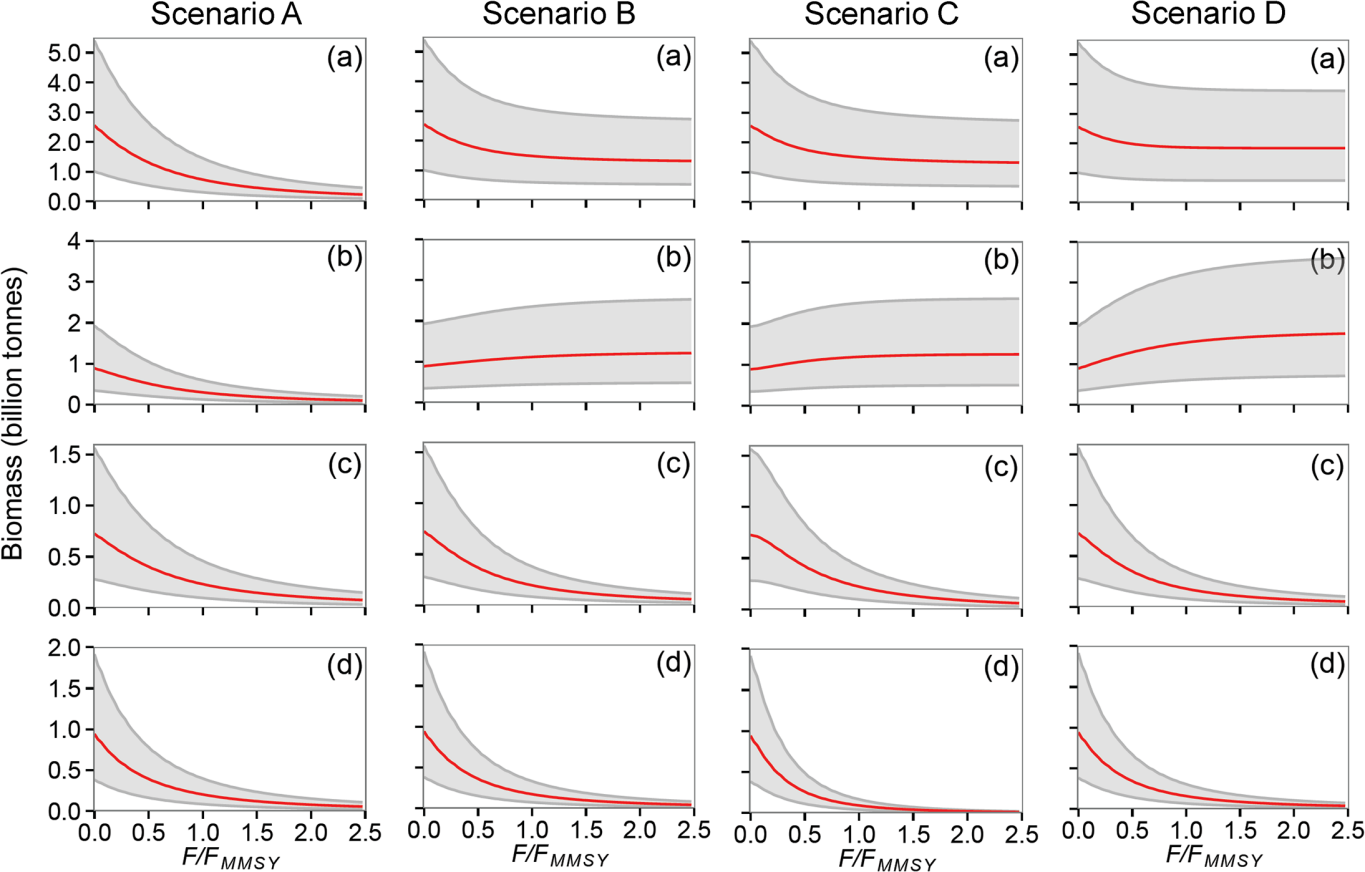
Predicted effects of fishing on global consumer biomass. Predicted changes in global biomass as a function of *F* (expressed as a proportion of *F*_*MMSY*_ for all consumers of body mass >100g) and selectivity. Each column presents results for one selectivity scenario (A to D, Table 3) and panels show biomass by consumer for (a) all individuals >100g (total), (b) 100g to 1 kg (small), (c) 1 kg to 10 kg (medium) and (d) > 10 kg (large).

Depletion of medium and large consumers at a given *F* is most extreme when fishing is less selective (Fig 7, scenario C, panels c and d). If small consumers are not targeted in FAO areas (Fig 7, scenarios B and C) then a slight increase in the global biomass of small consumers is predicted owing to prey release. Total depletion of all consumers at MMSY is predicted to be about 2/3 when small consumers are targeted globally (Fig 7, scenario A) but less than 50% when small consumers were not targeted (Fig 7, scenario D) or targeted only in the LME (Fig 7, scenario B, C). Predicted depletion of large consumers is much greater (> 25% at MMSY) if there is little separable control of *F* (Fig 7, scenario C). When smaller individuals are not heavily selected (scenario D), or selected in LME only (scenario B), median yields are lower. Under the assumption that individuals from 7cm could be targeted in LME only (scenario C) or in both LME and FAO areas (individuals from 8cm, scenario A) much higher yields result.

**Fig 8:**
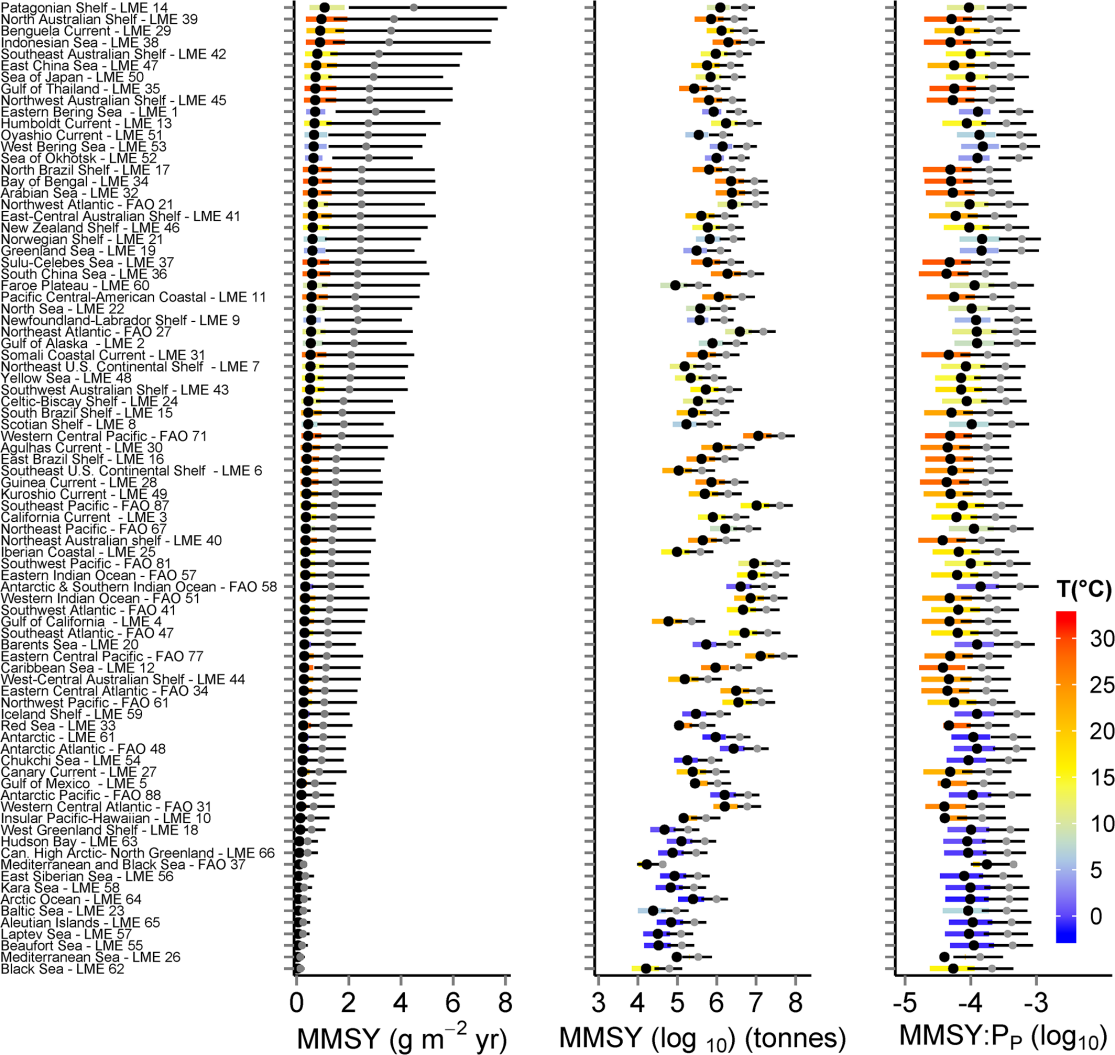
Predicted effects of selectivity on maximum multispecies sustainable yield. Estimated MMSY per unit area (left panel), absolute yield (middle panel) and the associated ratio of MMSY to *P*_*P*_ (right panel) by LME and FAO area for selectivity scenarios A and D. For selectivity scenario D, black points represent median estimates and coloured bars (mapped to sea surface temperature) the 25^th^ to 75^th^ percentiles. For selectivity scenario A, grey points represent median estimates and fine black bars the 25^th^ to 75^th^ percentiles. For corresponding figures for each individual selection scenario see S6–S9 Figs. Uncertainty intervals indicate the effects of parameter uncertainty in the macroecological model that was used to generate unexploited biomass estimates. They do not indicate uncertainty in underlying primary production estimates or uncertainty resulting from the structure and parameterisation of the size- and trait-based model.

Scenarios A and D assume the same selection pattern in all LME and FAO areas to allow comparison of predicted yields among these areas. On a per unit area basis, and with a focus on targeting of individuals >20cm (Fig 8, scenario D) highest median MMSY in any LME or FAO area just exceeds 1g m^-2^ yr^-1^ and uncertainty based on the 25^th^ and 75^th^ percentiles for unexploited biomass can just exceed ± 1g m^-2^ yr^-1^. These uncertainty ranges only reflect uncertainty in the unexploited biomass used to tune *κ* and not in other aspects of the parameterisation or structure of the size- and trait- based model. If smaller individuals are targeted (scenario A) the corresponding maximum MMSY increases to over 4 g m^-2^ yr^-1^. The most productive areas are predominantly temperate and tropical systems, with the Patagonian shelf, north Australian shelf and Benguela Current predicted to be the most productive areas, although some cool temperate systems have yield approaching 1 g (Fig 8, scenario D). Generally, MMSY per unit area is predicted to be lowest in some of the sub-polar and polar systems and deep enclosed seas and MMSY is heavily influenced by the selectivity scenario (S6–S9 Figs). Median consumer MMSY varies around 0.01% of primary production for selectivity scenario D and it is not systematically related to MMSY. Uncertainty in individual estimates of the ratio based on the 25^th^ and 75^th^ percentiles for unexploited biomass is ≈ 6 fold (Fig 8). The ratio of primary production to MMSY increases when smaller consumers are selected (scenarios A, B and C, S6–S9 Figs). Consumer MMSY is predicted to vary around 0.01% of primary production for selectivity scenario A and is not systematically related to MMSY (Fig 8). The ratio increases when smaller consumers are selected (scenarios A, B and C, S6–S9Figs).

## Discussion

Simple models based on a few established ecological principles and with minimal parameter demands provide insight into potential uncertainties in consumer biomass estimates that cannot be addressed with more complex models, especially when they have many tuned parameters. While these simple models do not include some structures or processes that can be important predictors of biomass distributions, especially on smaller scales, the underlying justifications for the structures of both models are founded in empirical and theoretical study of size-based processes in marine systems that show how body size, energy acquisition and transfer and the effects of temperature account for much of the variation in the structure and function of many types of communities [21, 26, 28, 29, 43, 48, 63, 64]. These simple approaches do not replace sophisticated and parameter hungry models for estimating population biomass, food web processes or fishing impacts at smaller scales (e.g. [10, 34–36, 65, 66]). But they do provide equitable treatment for large-scale system comparisons that include areas where the data needed to structure and parameterise more complex models are sparse and achieving better understanding of uncertainties is most pressing; thus allowing global questions to be addressed at global scales.

Uncertainties in TE, and to a lesser extent PPMR, account for most of the uncertainty in consumer biomass estimates. Existing sensitivity analyses have demonstrated the consequences of inaccurately estimating TE [11, 14] but based on recent data compilations [8, 16] our estimate of overall TE used in previous global production models [11] was close to the lower bound of possible values and thus resulted a mean consumer biomass estimate close to the lower bound of the current uncertainty intervals. Two other consumer biomass estimates [1, 12] fall within our uncertainty intervals, but the recent biomass estimate of 167 g m^-2^ [13] for marine consumers of 10^−5^ to 10^6^ g body mass was just over 4 fold higher than our median estimate of 38.6 g m^-2^ (1.4 × 10^10^ tonnes total) and 40 g m^-2^ higher than the 95^th^ percentile of our distribution of estimates (127 g m^-2^). A recent acoustic biomass estimate for mesopelagic fishes in the area from 40°N to 40°S also led to high median biomass estimates of 11 to 15 billion tonnes. Accompanying model estimates based on TE of 5–20% and 70–90% of primary production entering food chain resulted in biomass estimates of 2.3 to 71.2 billion tonnes for mesopelagic fishes [14]. For the area of the ocean from 40°N to 40°S, our macroecological model predicts median biomass for all consumers (assumed to include mesopelagic fishes) of 1.4 billion tonnes with a 95^th^ percentile of 8.1 billion tonnes, so even after accounting for uncertainty our median estimate is lower than their minimum estimate and our upper estimate below their median. The combination of wide uncertainties in our estimates and divergent values produced by different models and approaches has significant implications when estimating the role of marine consumers in biogeochemical cycles and supporting fisheries or predators. The main differences between approaches seem to be linked to the treatment of energy transfer in the first step in the food chain. Notwithstanding uncertainty linked to any assumed process, a stronger focus on understanding and representing low trophic level processes will be needed to help reconcile the divergent biomass estimates. For example, if it is assumed that almost 75% of primary production is grazed in tropical waters and gross growth efficiency reaches 30% [67] then the resulting TE of 22.5% is close to the upper values reported in compilations of TE used to estimate uncertainty [8, 16]. There is also the consideration that mesopelagic fishes have exceptionally high proximate lipid concentration [68] and relatively low metabolic demands per unit mass [69], with the consequence that our model that assumes average energy demands will tend to underestimate their mass while overestimating mass of pelagic species with higher energy demands.

The parameterisations of the macroecological model were reasonably supported by TE and PPMR estimates compiled in the last 5 years [8, 16, 48]. However, the majority of TE estimates come from the same types of model [8, 16] and efforts to collect and compile estimates from other sources would provide a valuable corroboration or challenge to our approach. For realised PPMR there remain inconsistencies between estimates from different sources and in the evidence for relationships between PPMR and consumer size. Existing PPMR estimates based on stable isotope methods [46, 70] may give near-complete coverage of all consumers in given body mass ranges but the ranges have been narrow (typically 3–4 orders of magnitude) in relation to the full size range of consumers (12+ orders of magnitude). Diet studies provide data for most of the full size range [26], but give incomplete coverage of individuals in a given body mass range and only provide a snapshot of diet (rather than stable isotope methods, which time-integrate information on the composition of assimilated diet).

Existing PPMR estimates based on stable isotopes suggest PPMR does not depend on consumer body mass but the absolute estimates of PPMR are quite variable. The recent proposal that nitrogen stable isotope fractionation is linked to prey *δ* ^15^ *N* [71], either directly or indirectly, has led to these data being revisited [72]. If fractionation does change with prey *δ* ^15^ *N* then preliminary analyses suggest that PPMR may be more consistent in different food webs then previously assumed; with variation in PPMR between studies falling from approximately 80 fold to 6 fold and a range in absolute PPMR of 49 to 316 [72]. These absolute values fall between the 5^th^ and 50^th^ percentiles of values simulated in the present study, but we also chose to take account of the higher PPMR recorded in diet studies when estimating variation in PPMR.

Diet studies, in contrast with the stable isotope studies, suggest that PPMR increases with consumer mass and even the 95^th^ percentile in simulations is low in relation to diet-based estimates of PPMR for individuals >10 kg body mass [48]. If this increase is real, then it is perhaps not surprising that it was not assumed or detected with stable isotope approaches when the deviations from linearity are relatively subtle over narrow size ranges and thus the potential to detect these deviations statistically would be low [48]. The macroecological model structure allows for the strength of the inverse relationship between TE and realised PPMR (S10 Fig) to be increased if future analyses of data support this. It is a trade-off that would lead to more consistency between size-spectrum slopes [48]. We conclude that existing studies of TE and PPMR do not lead to consistent outcomes and a priority for future work should be to refine estimates of TE and PPMR and understand interactions and links to the environment. This would contribute to large reductions in uncertainty in biomass predictions when applying the macroecological model. Even in the size- and trait-based model [32, 33], and other models of this type where TE is an output resulting from the processes contributing energy transfer, there are few data and many uncertainties surrounding the approaches needed to model the processes of prey encounter and capture. These processes include the probability of encountering prey and the probability of prey capture as a function of prey size and abundance.

The biomass of consumers predicted by the size-based models will include zooplankton, fish, squids, marine mammals and other groups. Despite tentative exploration of approaches that might allow fish to be distinguished [11, 54] we did not attempt such separation in this study and focused on consumers as a group. However, it is likely that the median biomass of fishes and squids exceeds 1.6 billion tonnes (the estimate of consumer biomass for individuals of 100g to 10 kg, with 5^th^ and 95^th^ percentiles of 0.1 and 8.9 billion tonnes) given that fish dominate biomass in this size-range and can have high relative abundance in other size-classes.

Predicted median maximum multispecies sustainable yield (MMSY) for medium-sized (maximum body mass 1–10 kg) and large species (> 10 kg) respectively ranged from 50 to 65 million tonnes and 19 to 26 million tonnes based on different gear selectivity. MMSY for fishes of all sizes depended primarily on the extent to which small fishes were assumed to be selected. Given the limited targeting of small fish outside a few LME where they are highly aggregated, then the models suggest that fisheries are unlikely to have reduced consumer biomass by > 1/3 globally, although this belies the depletion of individual stocks that are not considered in this analysis and the much greater depletion of medium and large consumers. If separation of mortality rates was weak then medium and large-sized consumers were fished well beyond their MMSY when MMSY was attained for the community. The marked depletion of consumers with body mass >1 kg will lead to a community with faster turnover times and more variable dynamics.

When smaller individuals are not heavily selected (scenario D), median global MMSY was closer to reported landings and discards (assuming 106 million tonnes yr^-1^ for 2010–2012, comprising 80 million tonnes reported landings [73], 18.5 million tonnes illegal or unreported landings (median estimate from [74]) and 8% discarding [75], although 106 million tonnes yr^-1^ should be treated as a minimum estimate given additional unreported landings from many coastal fisheries e.g. [76]). The quadrupling of predicted MMSY when smaller individuals (< 20 cm from species of maximum mass < 1kg) are targeted and when selection on larger species is more generalised shows how strongly t targeting of small individuals and species influences fisheries potential. Although predictions suggest that a shift to targeting smaller individuals of species with smaller maximum body size could markedly increase sustainable global yields this yield would not be accessible in most regions in practice. Reasons include (i) technical, economic, societal and political barriers to exploiting sparsely distributed but collectively abundant resources (e.g. mesopelagic fishes, krill), (ii) the marked effects of environmental variation on many small species, and (iii) the barriers to achieving independent control of fishing mortality on individuals of different sizes and species, which would otherwise provide higher yields while avoiding population collapse of larger and more sensitive species.

The presentations of global yield curves and biomass changes in response to fishing are novel, but sum responses in all LME and FAO areas. So, in scenarios where small species are targeted in the LME and not in the FAO areas, the global picture masks depletion of small species in the LME. These hypothetical comparative scenarios are suitable for comparing potential yield among areas, but are unlikely to provide reliable predictions of realised yields within areas. This is because there will be significant variation in size selectivity and fishing intensity between LME and FAO areas, as driven by social and cultural preferences, market demands, capacity or wish for selective fishing as well as differences in access, fisheries control and enforcement.

We do not attempt a systematic comparison with catches at the LME and FAO area scale because our results are intended to support comparisons of potential yield among areas based on consistent fishing scenarios and are not tailored to region specific fishing mortality or selectivity. Data on mortality and selectivity will not be available for the majority of areas in any case. However, for some upwelling regions (e.g. Humboldt) we note that our estimate of potential yield is lower than reported landings. This may reflect properties of food chains in upwellings, and the effective increase in TE for the whole food chain that results from the considerable plasticity of small pelagic fishes to select prey from a wide range of size classes and trophic levels, including primary producers, as their availability varies [77]. On the coastal margins of seas with low phytoplankton production our predicted yield is also lower than reported landings (e.g. Mediterranean Sea). This likely reflects the intensive targeting of small species and individuals by real fisheries in some of these areas. It also reflects underestimates of catch potential in the inshore zone, because the model does not account for primary production by groups other than phytoplankton (e.g. seagrass, macroalgae). This production may contribute 10% to global marine primary production, but accounts for a larger proportion of primary production close to coasts [24]. In most other areas, especially offshore, our yield predictions are higher than catches, likely reflecting that the development of fisheries for small species is primarily limited to a few areas where forage fishes form dense shoals. More widely dispersed small species are rarely targeted.

Although our approach addresses parameter uncertainty other caveats must be considered when interpreting our results. Briefly, inputs from the GCM are uncertain and there are differences between and within GCM and ocean color estimates of temperature and primary production. For example, a review of ocean color estimates of primary production highlighted variation of up to 83% between primary production estimates for individual ocean basins and even higher variation in the Arctic, Southern Ocean and Mediterranean (e.g. [23]). In some enclosed seas and coastal regions (e.g. Baltic Sea, Mediterranean Sea) the GCM estimates of primary production are much lower than those based on remote sensing and other sources of data. Thus higher estimates of biomass, production and potential yield for these seas and regions would be obtained with alternate primary production inputs. Our analysis of uncertainty did not address uncertainty in primary production inputs. In regions where stable consumer biomass estimates were not achieved with the size- and trait-based model, the GCM predicted that primary production was lower (S2 Table) than the primary production estimated from remote sensing and other sources of data. Exploratory analyses demonstrated that stability could be achieved if primary production was increased. However, we chose to model all regions with the same GCM inputs in this analysis, rather than make adjustments based on regional information. In future, it would be worthwhile repeating our approaches using multiple GCM and ocean color estimates of primary production, as well as regional data from other sources, to assess the variation in biomass estimates that results. Further, and as already mentioned in relation to potential fisheries yield, a model driven by pelagic primary production provides minimum estimates of consumer biomass and production because it ignores primary production by microphytobenthos, coral reef algae, macroalgae, seagrasses, marsh plants and mangroves. This primary production may support short and efficient food chains such as the direct grazing of turf algae on coral reefs.

Surface temperatures were used as proxy for thermal experience of consumers but distribution of biomass in relation to temperature varies in space and time. Our assumption leads to overestimates of individual production and mortality and underestimates of biomass, as surface waters are almost always warmer than the deeper waters. Although biomass in the deep sea is a very small proportion of that in pelagic waters and on shelves, the mean temperature experienced by abundant groups such as the mesopelagic fishes will be reduced by their use of cooler waters for all or part of the diurnal cycle. A future refinement to our approach would be to take account of, or seek to predict, the distribution of biomass in the water column and to link this to temperature at depth. Our model does not incorporate movement and so the fine-scale spatial distributions of production will be increasingly inaccurate for consumers of larger body mass [78].

In the real ocean marine mammals would contribute substantially to biomass in the largest size-classes. The biomass of marine mammals supported by a given rate of primary production will be overestimated in our modelling framework because their metabolic rates will be higher than rates predicted from water temperature. This biomass overestimate will be countered by a biomass underestimate that results from the high levels of low trophic level production that these animals often access by feeding at greatly elevated PPMR. These issues are not readily addressed in this simplified size-based modelling framework. If they were to be incorporated in size-based models their incorporation would be better supported in size- and species- based regional models that allow separate feeding and growth parameters to be defined and allow for large recruits to enter the size-spectrum (e.g.[34]). These models would be more appropriate for estimating the abundance and distribution of groups of ecological, conservation and fisheries interest.

Our analysis of the effects of fishing only accounted for uncertainty in unexploited biomass and not for structural or parameter uncertainty in the size- and trait-based model. It would be desirable to address the latter, as even in this elegant and relatively simple model several parameters cannot be directly estimated and uncertainty cannot currently be quantified. This is a recognised challenge when assessing uncertainty in a range of community and ecosystem models [79, 80].

In the size- and trait- based model we made some changes from previous parameterisations [33] to ensure persistence of all species when the model was run without fishing in all environments and to give plausible rates and trajectories of individual and population growth for all observed combinations of temperature and primary production ([33], S1 Table). In some cases these changes led to parameter values that took them outside ranges likely to be supported by data (e.g. sources cited in [33]), especially at low temperatures. This warrants further investigation and may be linked to incomplete specification of temperature effects and over-simplified functional forms (e.g. ecological adaptations to the environment that are not captured in the model).

Other uncertainties we did not consider include changes in euphotic depth driven by suspended particulate matter, especially in coastal regions (although in mixed and turbid coastal regions our use of mixed layer depth rather than euphotic depth will help to ameliorate this), and uncertainties in values of constants such as the remineralisation coefficient and carbon to wet weight conversions. These will all add additional uncertainty but are expected to have a small effect on biomass and production in relation to uncertainty driven by TE and PPMR. Our approach for addressing uncertainty also generates predictions in the tails of distributions that could be discounted for other reasons. For example, we did not reject size-spectrum slopes that were outside empirical ranges or estimates of production that were less than or equal to catches. Such constraints and refinements could be considered and included in future.

Perhaps the most remarkable finding to emerge from predictions of consumer abundance in the global oceans is the insight it provides into the relative scarcity of macroscopic animal life. Our median wet weight biomass estimate for consumers >1g body mass (Table 4) equates to 4.9 × 10^9^ km^3^ of animal tissue (if we make the simplifying assumption that consumer density is the same as that of seawater). This volume is smaller than that of a freshwater lake such as Loch Ness (7.4 km^3^), the largest and likely the best known of the freshwater lakes in the United Kingdom. Taking the volume of the global oceans as 1335 million km^3^ [81], the estimated volume of consumers >1g body mass implies that only one part by volume in 274 million is living macroscopic animal tissue. Stocking densities in intensive aquaculture, as one accessible comparison, would typically exceed 1 part in 100 by volume of fish. Even if we take as an extreme the upper uncertainty interval for the biomass estimate for consumers >10^−5^ g body mass (4.6 × 10^10^ tonnes), only one part in approximately 30 million by volume is living animal tissue. Thus the apparently high densities of marine life seen in surface and coastal waters and frequently visited abundance hotspots likely give many in society a false impression of the abundance of macroscopic marine animals; even though these animals still contribute substantially to some biogeochemical cycles and to global food security.

Overall, our analyses help to reconcile some of the existing global biomass estimates and highlight priorities for future analyses of the processes linking primary production and consumer production. If the model is sufficiently realistic to allow us to draw conclusions about the links between primary production and potential fisheries yield, it supports previous conclusions that efforts to link primary production and catches at global scales are unlikely to be successful, owing to differences in energy transfer processes at the base of the food chain [23]. Our results also provide a set of baselines for assessing human impacts and predicting the contribution of marine animals to biogeochemical processes. Since our model assumes that the same structure and parameterisation can be adopted in all regions, it will likely serve as a useful null model for comparison with more complex regional models

## Supporting Information

S1 Fig Predicted effects of temperature and primary production on consumer biomass and production. (PDF)

S2 Fig Global distributions of consumer biomass and production. (PDF)

S3 Fig Uncertainty in consumer biomass and production by LME and FAO areas. (PDF)

S4 Fig Median MMSY estimates by body mass with selection scenario C. (PDF)

S5 Fig Total median MMSY estimates by LME for the four selection scenarios. (PDF)

S6 Fig Uncertainty in MMSY estimates by LME and FAO areas with selectivity scenario A. (PDF)

S7 Fig Uncertainty in MMSY estimates by LME and FAO areas with selectivity scenario B. (PDF)

S8 Fig Uncertainty in MMSY estimates by LME and FAO areas with selectivity scenario C. (PDF)

S9 Fig Uncertainty in MMSY estimates by LME and FAO areas with selectivity scenario D. (PDF)

S10 Fig Simulated relationship between the predator prey mass ratio and trophic transfer efficiency. (PDF)

S1 Table Parameter values for the size and trait-based model. (PDF)

S2 Table Environmental properties, areas and codes for LME and FAO areas. (PDF)
