# Supplementary material for: Predicting Consumer Biomass, Size-Structure, Production, Catch Potential, Responses to Fishing and Associated Uncertainties in the World’s Marine Ecosystems
Source: PLoS One. 2015 Jul 30;10(7):e0133794. doi: 10.1371/journal.pone.0133794 (PMC4520681; doi:10.1371/journal.pone.0133794)
Supplement: S1 Fig — (PDF) [file pone.0133794.s001.pdf]

S1 Fig.

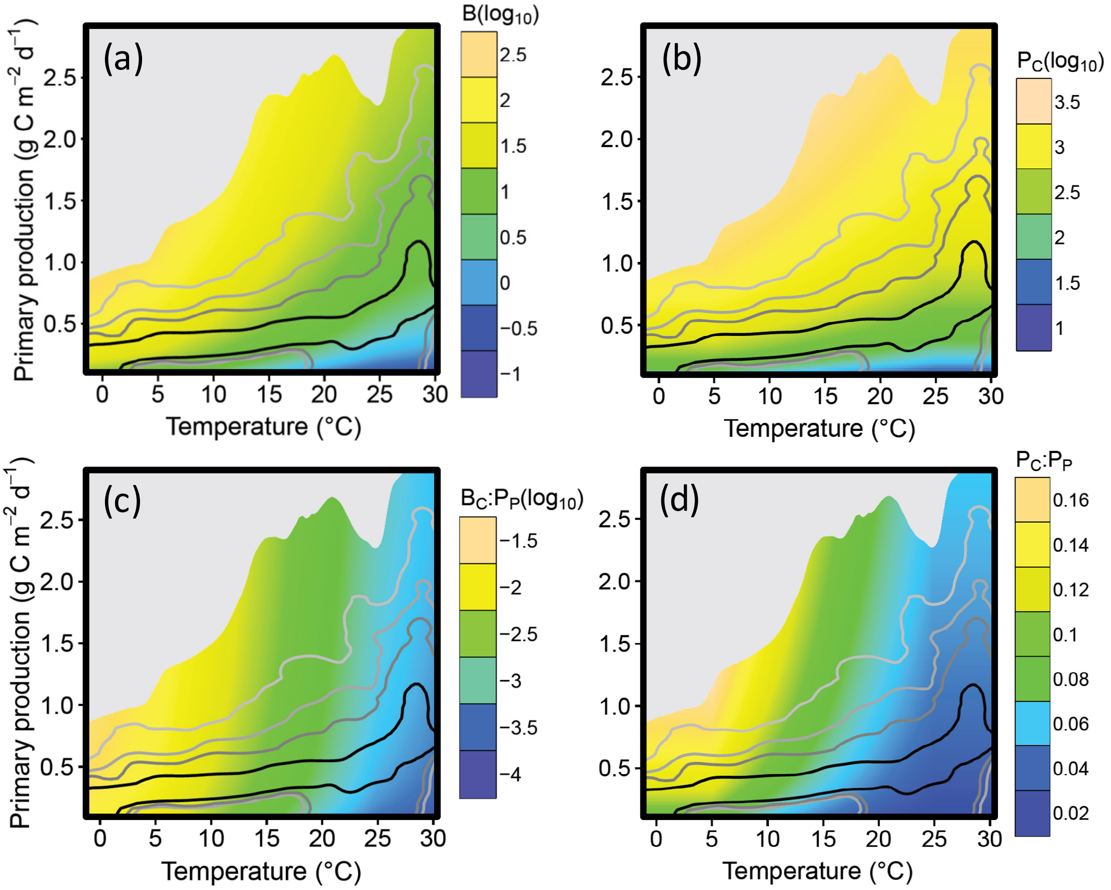

S1 Fig. Predicted effects of temperature and primary production on consumer biomass and production. Modelled relationships between temperature T_C and daily primary production P_P and (a) median estimated consumer biomass B (g m^{-2} in M range 10^2 to 10^4 g), (b) median estimated consumer production P_C (g m^{-2} yr^{-1} in M range 10^2 to 10^4 g), (c) ratio of primary production (g m^{-2} yr^{-1}) to consumer biomass B_C (g m^{-2} in M range 10^2 to 10^4 g) and (d) ratio of primary production (g m^{-2} yr^{-1}) to consumer production P_C (g m^{-2} yr^{-1} in M range 10^2 to 10^4 g). In all simulations Z_e was assumed to be 50 m and Z 200 m. Chlorophyll concentration was estimated from primary production using a relationship established from the GCM outputs. Values of T_C and P_P that fell outside ranges including 99.99% of GCM outputs for the world's oceans are masked. Contours indicate combinations of T_C and P_P that include 70% (black), 90%, 95% and 99% (pale grey) of GCM outputs.
