# Supplementary material for: Predicting Consumer Biomass, Size-Structure, Production, Catch Potential, Responses to Fishing and Associated Uncertainties in the World’s Marine Ecosystems
Source: PLoS One. 2015 Jul 30;10(7):e0133794. doi: 10.1371/journal.pone.0133794 (PMC4520681; doi:10.1371/journal.pone.0133794)
Supplement: S2 Fig — (PDF) [file pone.0133794.s002.pdf]

S2 Fig.

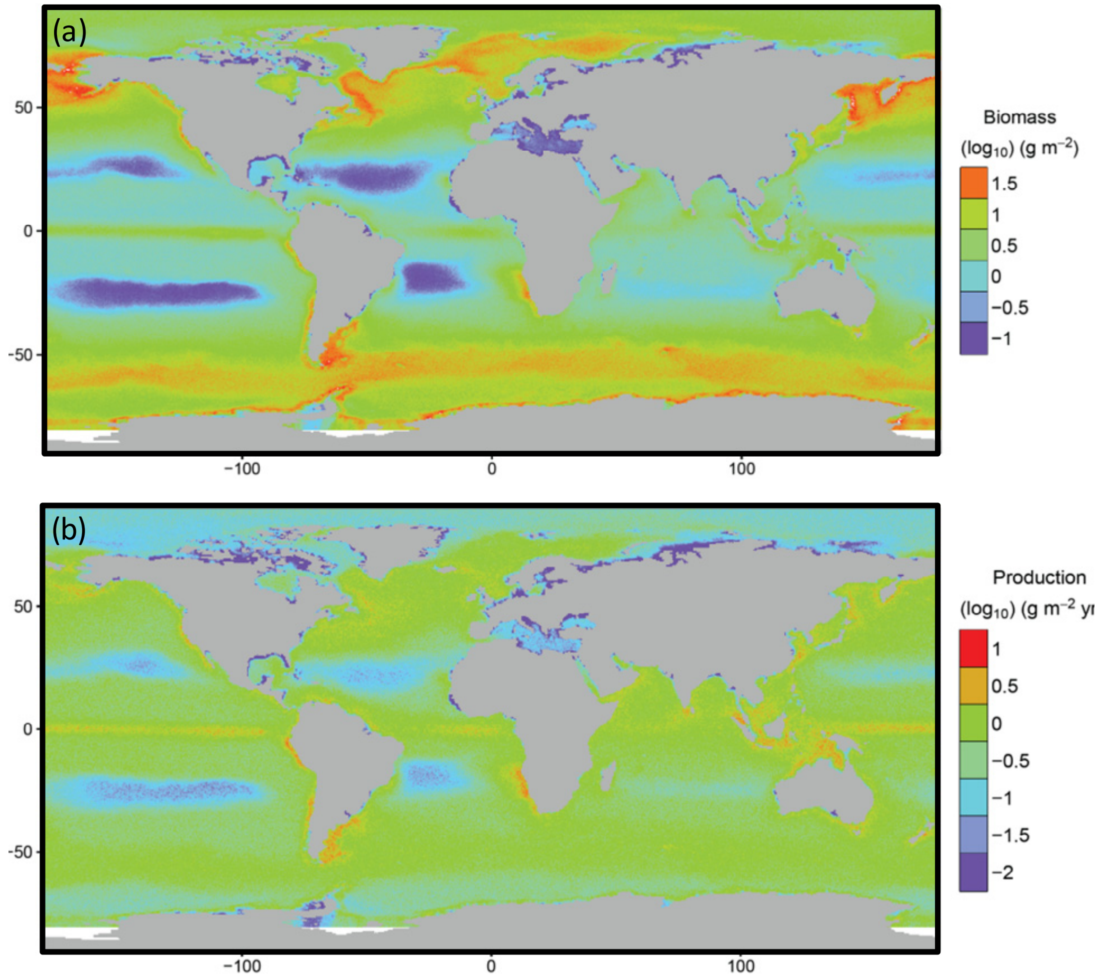

S2 Fig. Global distribution of consumer biomass and production. Predicted global distribution of (a) consumer biomass, (b) production and (c) the consumer production: biomass ratio for individuals of body mass 10^2 to 10^4 g. Areas in white, predominantly in the Southern Ocean, are marine areas not included in the GCM domain.

This page is a supplement from "Predicting consumer biomass, size-structure, production, catch potential, responses to fishing and associated uncertainties in the world's marine ecosystems" Jennings and Collingridge, PLoS One.
