# Supplementary material for: Predicting Consumer Biomass, Size-Structure, Production, Catch Potential, Responses to Fishing and Associated Uncertainties in the World’s Marine Ecosystems
Source: PLoS One. 2015 Jul 30;10(7):e0133794. doi: 10.1371/journal.pone.0133794 (PMC4520681; doi:10.1371/journal.pone.0133794)
Supplement: S3 Fig — (PDF) [file pone.0133794.s003.pdf]

S3 Fig.

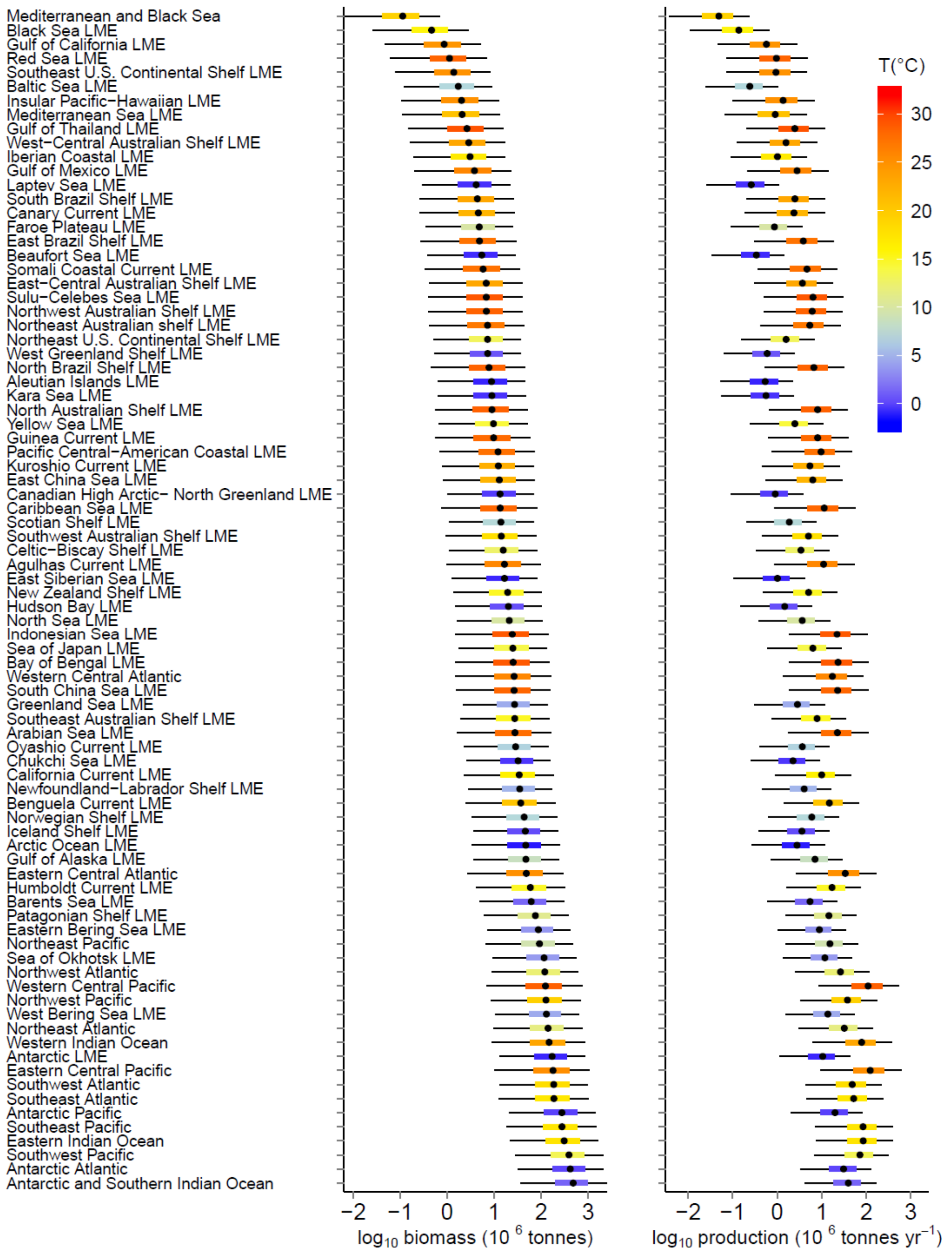

S3. Fig. Uncertainty in consumer biomass and production by LME and FAO areas. Estimates of total consumer biomass and total consumer production by LME and FAO areas as predicted with the macroecological model for consumers of 1g to 10^6 g. Points represent median production estimates, coloured bars (mapped to Sea Surface Temperature) the 25th to 75th percentiles and black lines the 5th to 95th percentiles. Uncertainty intervals show the effects of parameter uncertainty in the macroecological model but do not account for structural uncertainty or uncertainty in primary production and temperature inputs.

This page is a supplement from “Predicting consumer biomass, size-structure, production, catch potential, responses to fishing and associated uncertainties in the world’s marine ecosystems” Jennings and Collingridge, PLoS One.
