# Supplementary material for: Predicting Consumer Biomass, Size-Structure, Production, Catch Potential, Responses to Fishing and Associated Uncertainties in the World’s Marine Ecosystems
Source: PLoS One. 2015 Jul 30;10(7):e0133794. doi: 10.1371/journal.pone.0133794 (PMC4520681; doi:10.1371/journal.pone.0133794)
Supplement: S4 Fig — (PDF) [file pone.0133794.s004.pdf]

S4 Fig.

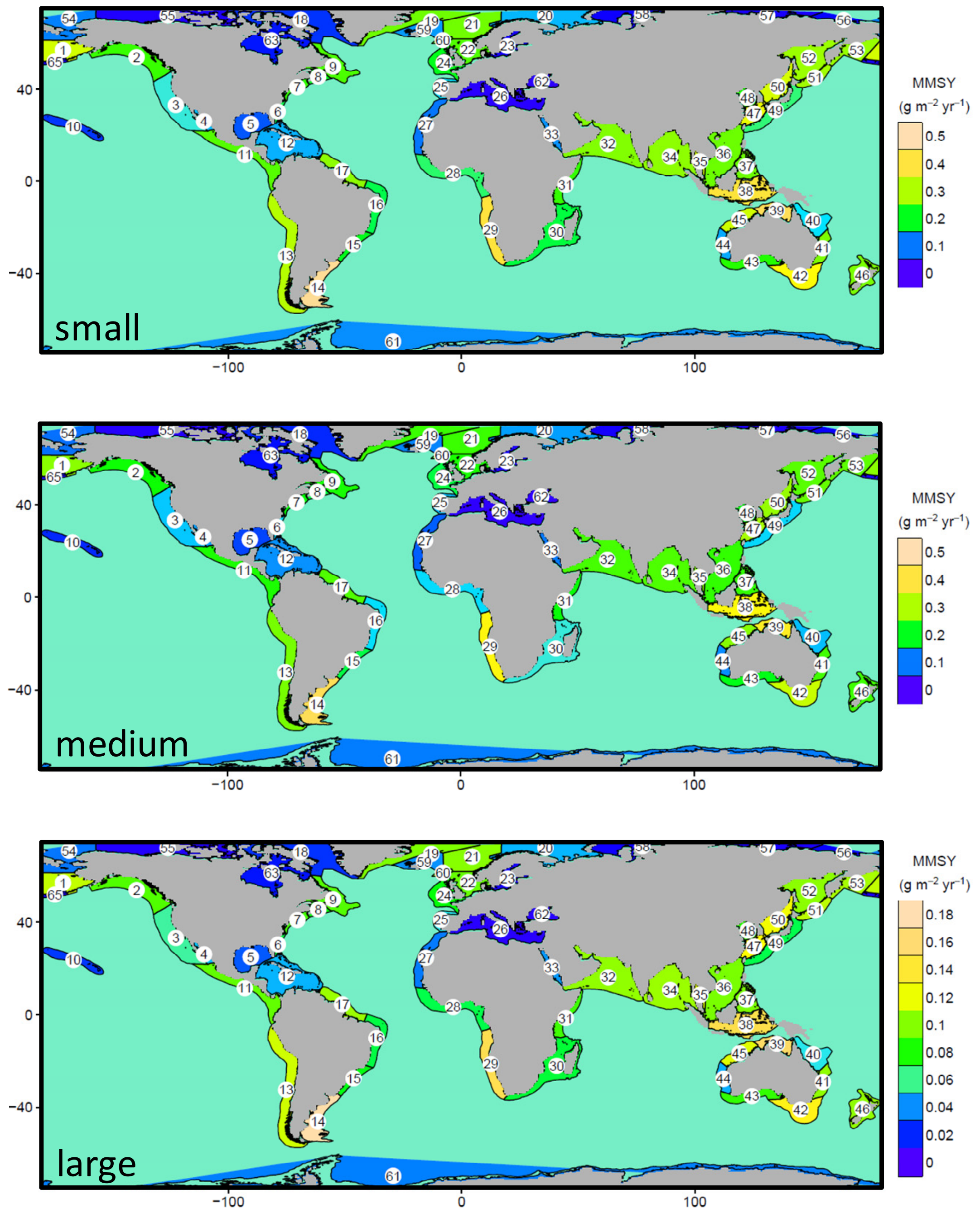

S4 Fig. Median MMSY estimates by body mass with selection scenario C. Median estimate of the predicted maximum multispecies sustainable yield by LME when fishing with selectivity Scenario C. Upper panel for small species (body mass <10^3g), centre panel for medium-sized species (10^3-10^4 g) and lower panel for large species (>10^4 g).

This page is a supplement from "Predicting consumer biomass, size-structure, production, catch potential, responses to fishing and associated uncertainties in the world's marine ecosystems" Jennings and Collingridge, PLoS One.
