# Supplementary material for: Predicting Consumer Biomass, Size-Structure, Production, Catch Potential, Responses to Fishing and Associated Uncertainties in the World’s Marine Ecosystems
Source: PLoS One. 2015 Jul 30;10(7):e0133794. doi: 10.1371/journal.pone.0133794 (PMC4520681; doi:10.1371/journal.pone.0133794)
Supplement: S5 Fig — (PDF) [file pone.0133794.s005.pdf]

S5 Fig.

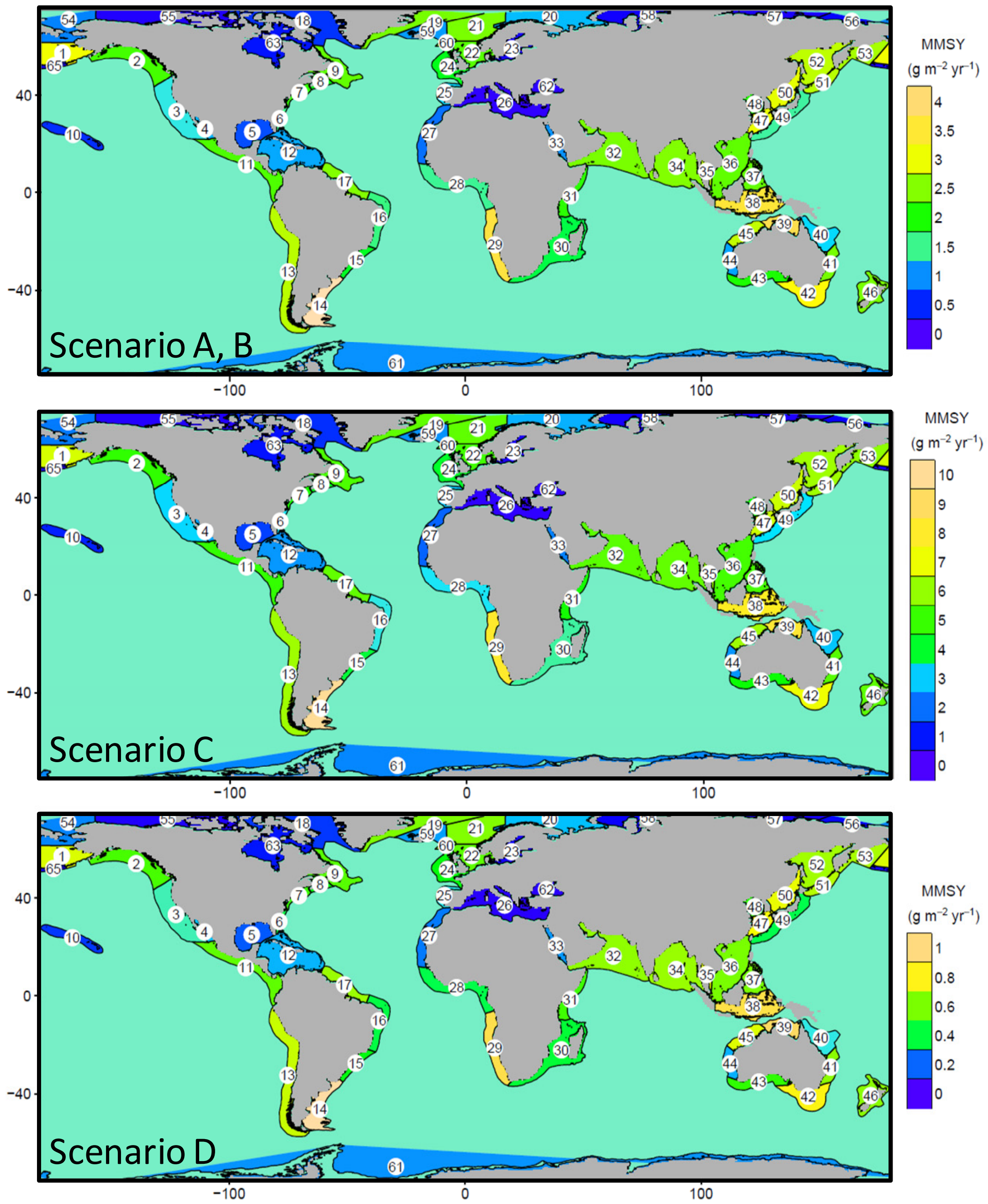

S5 Fig. Total median MMSY estimates by LME for the four selection scenarios. Median estimate of the predicted maximum multispecies sustainable yield from species of all sizes in all LME when fishing with selectivity Scenarios A and B, C or D.

This page is a supplement from "Predicting consumer biomass, size-structure, production, catch potential, responses to fishing and associated uncertainties in the world's marine ecosystems" Jennings and Collingridge, PLoS One.
