# Supplementary material for: Predicting Consumer Biomass, Size-Structure, Production, Catch Potential, Responses to Fishing and Associated Uncertainties in the World’s Marine Ecosystems
Source: PLoS One. 2015 Jul 30;10(7):e0133794. doi: 10.1371/journal.pone.0133794 (PMC4520681; doi:10.1371/journal.pone.0133794)
Supplement: S8 Fig — (PDF) [file pone.0133794.s008.pdf]

S8 Fig.

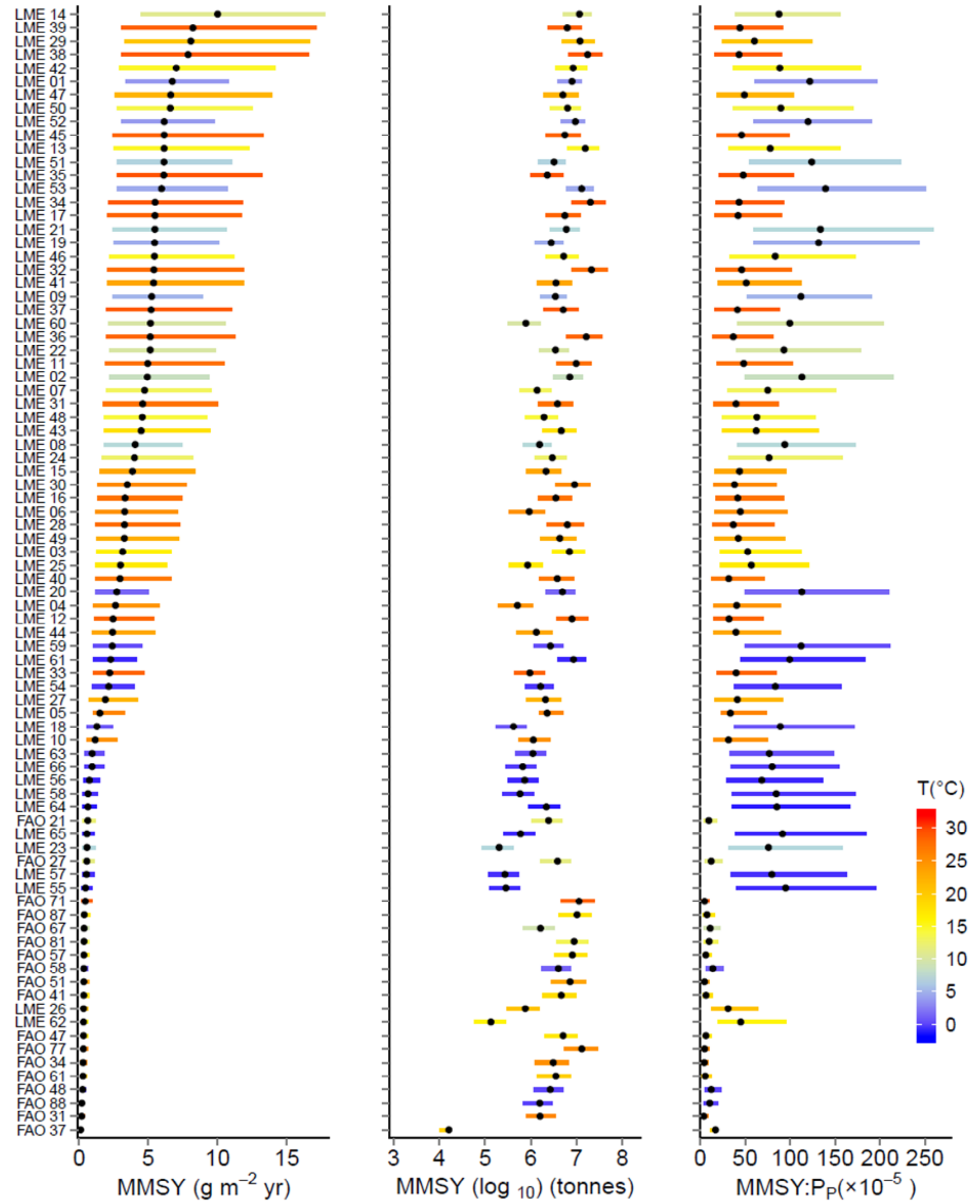

S8 Fig. Uncertainty in MMSY estimates by LME and FAO areas with selectivity scenario C. Estimated maximum multispecies sustainable yield (MMSY) by LME and FAO areas and the associated ratio of consumer production (CP) to primary production (PP) assuming selectivity scenario C. Horizontal lines span outputs from model runs based on the 25th to 75th percentiles for unexploited biomass and points show the 50th percentile. Sea surface temperatures (SST) are mapped to the horizontal lines. Uncertainty intervals indicate the effects of parameter uncertainty in the macroecological model that was used to generate unexploited biomass estimates. They do not indicate uncertainty in underlying primary production estimates or the structure and parameterisation of the size- and trait-based model

This page is a supplement from "Predicting consumer biomass, size-structure, production, catch potential, responses to fishing and associated uncertainties in the world's marine ecosystems" Jennings and Collingridge, PLoS One.
