# Supplementary material for: Predicting Consumer Biomass, Size-Structure, Production, Catch Potential, Responses to Fishing and Associated Uncertainties in the World’s Marine Ecosystems
Source: PLoS One. 2015 Jul 30;10(7):e0133794. doi: 10.1371/journal.pone.0133794 (PMC4520681; doi:10.1371/journal.pone.0133794)
Supplement: S10 Fig — (PDF) [file pone.0133794.s010.pdf]

S10 Fig.

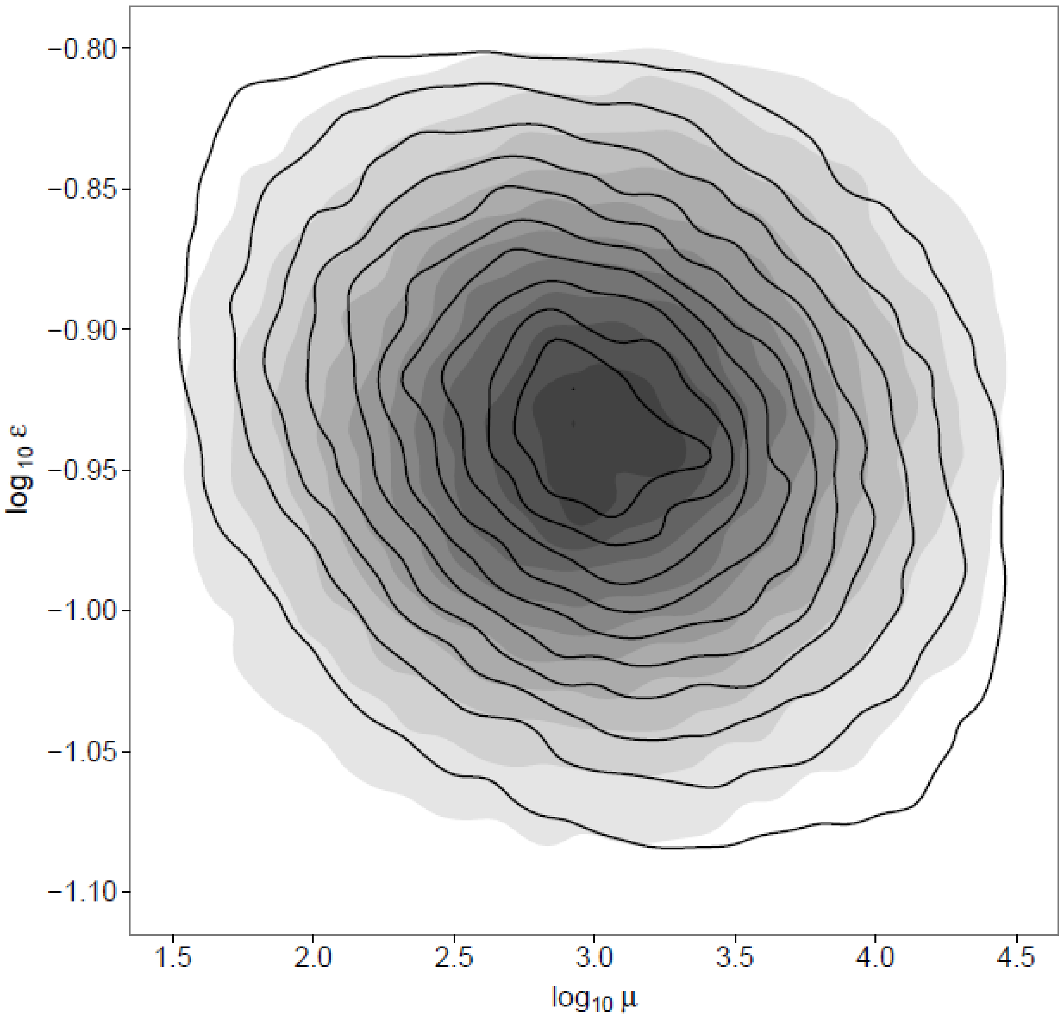

S10 Fig. Simulated relationship between the predator prey mass ratio and trophic transfer efficiency. Simulated relationship between the predator prey mass ratio (μ) and trophic transfer efficiency (ε). Black contour lines enclose 10-100% of estimated values (in 10% increments) from 10000 draws while shaded contours show the results from 10000 draws where no relationship between μ and ε was assumed (also in 10% increments from 10-100%).
