# Supplementary material for: Predicting Consumer Biomass, Size-Structure, Production, Catch Potential, Responses to Fishing and Associated Uncertainties in the World’s Marine Ecosystems
Source: PLoS One. 2015 Jul 30;10(7):e0133794. doi: 10.1371/journal.pone.0133794 (PMC4520681; doi:10.1371/journal.pone.0133794)
Supplement: S1 Table — (PDF) [file pone.0133794.s011.pdf]

S1 Table. Parameter values for the size and trait-based model. Parameter values for the size and trait-based model. Parameters and symbols follow descriptions in Hartvig *et al*. [1]. Calibrated parameters assume different values depending on biomass estimates generated by the macroecological model. Parameters are marked "variable" if they were modified to account for differences in temperature and/or primary production among systems. Efficiency of offspring production (ε in [1]) was significantly reduced to account for early life mortality [2]. When parameter values were variable the base value is given.

**Parameter**	**Description**	**Value**
Individual growth	initial feeding level (f_0 in [1])	0.45
	assimilation efficiency (α in [1])	0.55
	maximum food intake (h in [1])	25 base value (variable)
	exponent maximum food intake (n in [1])	0.67
	standard metabolism and activity (k in [1])	2.5 base value (variable)
	exponent of standard metabolism (p in [1])	0.75
	preferred predator-prey mass ratio (β in [1])	580
	width of feeding kernel (σ in [1])	2.7
	exponent of search volume[1] (σ in [1])	0.9
Reproduction	offspring mass (g) (m_0 in [1])	0.005
	mass at maturity as fraction of asymptotic mass (η in [1])	0.25
	efficiency of offspring production (ε in [1])	0.01
	width of maturation transition (u in [1])	10
Mortality	fraction of energy reserves (ξ in [1])	0.1
	background mortality (μ_0 in [1])	0.5 base value (variable)
Resource spectrum	magnitude of resource spectrum (κ in [1])	calibrated (variable)
	slope of resource spectrum ($2-n+q$ in [1])	2.23
	regeneration of resource spectrum (r_0 in [1])	calibrated (variable)
	upper size limit of resource spectrum (g) (m_{cut} in [1])	0.1

References

1. Hartvig M, Andersen KH, Beyer JE 2011. Food web framework for size-structured populations. Journal of Theoretical Biology, 272,113-122.

2. Bailey KM, Houde ED 1989. Predation on eggs and larvae of marine fishes and the recruitment problem. Advances in Marine Biology 25, 1-83.
