# Supplementary material for: Predicting Consumer Biomass, Size-Structure, Production, Catch Potential, Responses to Fishing and Associated Uncertainties in the World’s Marine Ecosystems
Source: PLoS One. 2015 Jul 30;10(7):e0133794. doi: 10.1371/journal.pone.0133794 (PMC4520681; doi:10.1371/journal.pone.0133794)
Supplement: S2 Table — (PDF) [file pone.0133794.s012.pdf]

S2 Table. Environmental properties, areas and codes for LME and FAO areas. Large Marine Ecosystems (LME) and FAO Fishing Areas included in the analysis. The FAO area calculations exclude areas that are allocated to LME. Temperature *T* and primary production P_P estimates are calculated from Global Circulation Model input data. Primary production estimates for individual LME and FAO areas will differ from estimates derived from remote sensing data or regional measurements and models.

name of LME or FAO area	code	area (10^6 km^2)	*T* (°C)	P_P (g C m^{-2} d^{-1})
Eastern Bering Sea LME	1	1.17	3.6	0.53
Gulf of Alaska LME	2	1.43	8.5	0.42
California Current LME	3	2.21	16.2	0.57
Gulf of California LME	4	0.19	24.7	0.62
Gulf of Mexico LME	5	1.51	25.9	0.43
Southeast U.S. Continental Shelf LME	6	0.28	24.9	0.71
Northeast U.S. Continental Shelf LME	7	0.29	13.1	0.61
Scotian Shelf LME	8	0.38	7.1	0.41
Newfoundland-Labrador Shelf LME	9	0.65	5.0	0.45
Insular Pacific-Hawaiian LME	10	0.98	25.1	0.35
Pacific Central-American Coastal LME	11	1.96	27.5	0.99
Caribbean Sea LME	12	3.22	28.2	0.74
Humboldt Current LME	13	2.51	15.0	0.76
Patagonian Shelf LME	14	1.15	10.8	1.10
South Brazil Shelf LME	15	0.55	23.1	0.85
East Brazil Shelf LME	16	1.06	27.3	0.77
North Brazil Shelf LME	17	1.01	28.4	1.25
West Greenland Shelf LME	18	0.33	0.6	0.14
Greenland Sea LME	19	0.51	4.5	0.40
Barents Sea LME	20	1.80	1.3	0.23
Norwegian Shelf LME	21	1.09	7.1	0.39
North Sea LME	22	0.67	9.9	0.53
Baltic Sea LME	23	0.36	6.9	0.07
Celtic-Biscay Shelf LME	24	0.74	12.5	0.50
Iberian Coastal LME	25	0.28	16.9	0.51
Mediterranean Sea LME	26	2.44	20.8	0.10
Canary Current LME	27	1.10	22.0	0.44
Guinea Current LME	28	1.91	28.0	0.86
Benguela Current LME	29	1.46	20.4	1.29
Agulhas Current LME	30	2.58	25.8	0.88
Somali Coastal Current LME	31	0.83	27.5	1.11
Arabian Sea LME	32	3.89	27.8	1.13
Red Sea LME	33	0.43	28.4	0.53
Bay of Bengal LME	34	3.63	29.1	1.22
Gulf of Thailand LME	35	0.37	29.4	1.23
South China Sea LME	36	3.14	28.2	1.35
Sulu-Celebes Sea LME	37	0.98	29.2	1.21
Indonesian Sea LME	38	2.21	29.1	1.76
North Australian Shelf LME	39	0.76	28.6	1.80
Northeast Australian shelf LME	40	1.28	26.9	0.90
East-Central Australian Shelf LME	41	0.66	23.2	1.02
Southeast Australian Shelf LME	42	1.19	15.1	0.76
Southwest Australian Shelf LME	43	1.03	17.9	0.69
West-Central Australian Shelf LME	44	0.54	23.1	0.59
Northwest Australian Shelf LME	45	0.90	28.6	1.29
New Zealand Shelf LME	46	0.96	15.1	0.63

S2 Table (continued)

**name of LME or FAO area**	**code**	**area (10^6 km^2)**	***T*** **(°C)**	**P_P (g C m^{-2} d^{-1})**
East China Sea LME	47	0.76	22.0	1.30
Yellow Sea LME	48	0.42	14.1	0.70
Kuroshio Current LME	49	1.31	22.5	0.74
Sea of Japan LME	50	0.96	13.7	0.71
Oyashio Current LME	51	0.52	6.6	0.48
Sea of Okhotsk LME	52	1.52	3.8	0.49
West Bering Sea LME	53	2.15	4.5	0.41
Chukchi Sea LME	54	0.76	-0.7	0.25
Beaufort Sea LME	55	0.63	-1.1	0.05
East Siberian Sea LME	56	0.99	-1.3	0.10
Laptev Sea LME	57	0.51	-0.9	0.06
Kara Sea LME	58	0.91	-0.8	0.07
Iceland Shelf LME	59	1.12	0.1	0.21
Faroe Plateau LME	60	0.15	9.8	0.50
Antarctic LME	61	3.75	-1.3	0.22
Black Sea LME	62	0.44	15.7	0.07
Hudson Bay LME	63	1.18	0.3	0.12
Arctic Ocean LME	64	3.48	-1.6	0.07
Aleutian Islands LME	65	1.07	-1.3	0.06
Canadian High Arctic- North Greenland LME	66	0.71	-0.5	0.11
Northwest Atlantic FAO area	FAO 21	3.95	12.1	0.63
Northeast Atlantic FAO area	FAO 27	6.96	11.5	0.43
Western Central Atlantic FAO area	FAO 31	9.14	25.8	0.43
Eastern Central Atlantic FAO area	FAO 34	11.18	25.1	0.61
Mediterranean and Black Sea FAO area	FAO 37	0.16	19.2	0.06
Southwest Atlantic FAO area	FAO 41	14.36	18.1	0.48
Southeast Atlantic FAO area	FAO 47	16.64	17.2	0.47
Antarctic Atlantic FAO area	FAO 48	10.91	-0.1	0.19
Western Indian Ocean FAO area	FAO 51	22.03	23.3	0.66
Eastern Indian Ocean FAO area	FAO 57	24.07	17.6	0.52
Antarctic and Southern Indian Ocean FAO area	FAO 58	12.05	1.2	0.23
Northwest Pacific FAO area	FAO 61	13.07	19.4	0.47
Northeast Pacific FAO area	FAO 67	4.62	9.3	0.31
Western Central Pacific FAO area	FAO 71	25.63	28.7	0.88
Eastern Central Pacific FAO area	FAO 77	43.90	25.3	0.59
Southwest Pacific FAO area	FAO 81	25.96	13.3	0.33
Southeast Pacific FAO area	FAO 87	27.95	17.3	0.47
Antarctic Pacific FAO area	FAO 88	8.50	-0.3	0.17
